# Evolution of *pogo*, a separate superfamily of *IS630-Tc1-mariner* transposons, revealing recurrent domestication events in vertebrates

**DOI:** 10.1186/s13100-020-00220-0

**Published:** 2020-07-22

**Authors:** Bo Gao, Yali Wang, Mohamed Diaby, Wencheng Zong, Dan Shen, Saisai Wang, Cai Chen, Xiaoyan Wang, Chengyi Song

**Affiliations:** College of Animal Science and Technology, Yangzhou University, Yangzhou, 225009 Jiangsu China

**Keywords:** *pogo*, DNA transposons, *Tc1/mariner*, *IS630*, Evolution, Domestication

## Abstract

**Background:**

*Tc1/mariner* and *Zator*, as two superfamilies of *IS630-Tc1*-*mariner* (*ITm*) group, have been well-defined. However, the molecular evolution and domestication of *pogo* transposons, once designated as an important family of the *Tc1/mariner* superfamily, are still poorly understood.

**Results:**

Here, phylogenetic analysis show that *pogo* transposases, together with *Tc1/mariner*, DD34E/*Gambol*, and *Zator* transposases form four distinct monophyletic clades with high bootstrap supports (> = 74%), suggesting that they are separate superfamilies of *ITm* group. The *pogo* superfamily represents high diversity with six distinct families (*Passer*, *Tigger*, *pogoR*, *Lemi*, *Mover*, and *Fot/Fot-like*) and wide distribution with an expansion spanning across all the kingdoms of eukaryotes. It shows widespread occurrences in animals and fungi, but restricted taxonomic distribution in land plants. It has invaded almost all lineages of animals—even mammals—and has been domesticated repeatedly in vertebrates, with 12 genes, including centromere-associated protein B (CENPB), CENPB DNA-binding domain containing 1 (CENPBD1), Jrk helix–turn–helix protein (JRK), JRK like (JRKL), *pogo* transposable element derived with KRAB domain (POGK), and with ZNF domain (POGZ), and *Tigger* transposable element-derived 2 to 7 (TIGD2–7), deduced as originating from this superfamily. Two of them (JRKL and TIGD2) seem to have been co-domesticated, and the others represent independent domestication events. Four genes (TIGD3, TIGD4, TIGD5, and POGZ) tend to represent ancient domestications in vertebrates, while the others only emerge in mammals and seem to be domesticated recently. Significant structural variations including target site duplication (TSD) types and the DDE triad signatures (DD29–56D) were observed for *pogo* transposons. Most domesticated genes are derived from the complete transposase genes; but CENPB, POGK, and POGZ are chimeric genes fused with additional functional domains.

**Conclusions:**

This is the first report to systematically reveal the evolutionary profiles of the *pogo* transposons, suggesting that *pogo* and *Tc1/Mariner* are two separate superfamilies of *ITm* group, and demonstrating the repeated domestications of *pogo* in vertebrates. These data indicate that *pogo* transposons have played important roles in shaping the genome and gene evolution of fungi and animals. This study expands our understanding of the diversity of *pogo* transposons and updates the classification of *ITm* group.

## Introduction

Transposable elements or transposons are viewed as molecular parasites and segments of genetic material that can ensure their own replication (albeit with the help of host factors). They are sometimes called “jumping genes” for their ability to jump around from place to place on chromosomes and are found in both prokaryotic and eukaryotic genomes [1, 2]. Based on the “jumping” mechanism, transposons are classified into RNA transposons (retrotransposons), which move using an RNA intermediate, along with reverse transcriptase to produce the complementary DNA, and DNA transposons, which move about using a DNA intermediate associated with a transposase [3]. Multiple transposition mechanisms of DNA transposons have been defined and they can be subdivided into three major types: the cut-and-paste, peel-and-paste, and self-synthesizing transposons [3–5].

Transposons are thought to have played important roles in the evolution of individual genes and in shaping the genomic landscape of their host [6, 7]. It has been suggested that transposons play important roles in genome size variations in vertebrates [8, 9], and they constitute a large fraction (30–50%) of mammal genomes [10]. It has been found that some DNA transposons can undergo “molecular domestication” a process through which they evolve new cellular functions but also lose their mobility due to loss of function of the two minimally required functional components: the terminal inverted repeat (TIR) sequences and the transposase [7, 11]. Many protein-coding genes in mammals have evolved from DNA transposons; about 50 domesticated genes in the human genome have been reported [7, 11], and these have derived from diverse DNA transposons, such as THAP9 derived from the *P* element [12], SETMAR derived from *Tc1/mariner* [13], RAG proteins derived from *Transib* [14], and PGBD5 derived from *piggyBac* [15].

*Tc1/mariner*, a superfamily of cut-and-paste transposons named after the first element identified in *Caenorhabditis elegans* (Transposon *C. elegans* number 1, *Tc1*) [16] and *Drosophila mauritiana* (*mariner*) [17] is thought to be the most widespread group of DNA transposons, and multiple distinct families (DD34D*/mariner*, DD37D*/maT*, DD39D, DD41D, DD34E*/Tc1*, DD35E*/TR*, DD36E*/IC*, and DD37E*/TRT*) of *Tc1/mariner* have been well-defined [18–23]. The eukaryotic superfamily *Tc1/mariner* is related to the bacterial *IS630* family [24], which is also referred to as the *IS630-Tc1-Mariner* (*ITm*) group [25–27]. *Zator* was identified as a superfamily and related to the bacterial *TP36* family of transposases [28]. However, *Zator* and *TP36* are also clustered with the bacterial *IS630* family, along with the *Tc1/mariner* [28], indicating that the *ITm* group represents high diversity and the phylogenetic relationship across these transposons is still waiting to be defined. The *pogo* element was firstly identified in flies [29], then diverse relative transposons including *Tigger* in humans [30], *Fot*, *Tan1*, *Pot1*, *Pot2*, *Flipper*, and *Aft1*transposons in fungi [31–36], *pogo*-like elements (*Lemi1*) in plants [37], and *pogo*-like elements in teleosts [38] have been identified and they were close to *pogo* transposase in phylogenetic position [9, 26, 38]. This group was named as DD × D*/pogo* [26], and it was believed to belong to the *Tc1/mariner* superfamily for long time [25, 26]. However, the origin, taxonomic distribution, diversity, and molecular domestication of the *pogo* transposons remain largely unknown. In addition, although the domestication of CENPB has been well characterized [39], the origins of several other related genes, including TIGD1–TIGD7, JRK and JRKL, are ambiguous [39, 40], and the evolutionary relationships between them remain unknown. Here, we systematically investigate the taxonomic distribution of *pogo* transposons, as well as their domestication in vertebrates, and characterize the phylogenetic relationships, structural organization, and conservation of these transposons and their domesticated proteins. Our data display, for the first time, the entire evolutionary landscape of *pogo* transposons and their domestication in vertebrates, and we also provide evidence to support that *pogo* is a separate superfamily and evolved independently from *IS630* transposases. These findings have important implications for understanding the evolution of the *pogo* transposons, as well as their impact on genome and gene evolution.

## Results

### *pogo* and *Tc1/Mariner* are two distinct superfamilies of *ITm* transposons

**Fig. 1:**
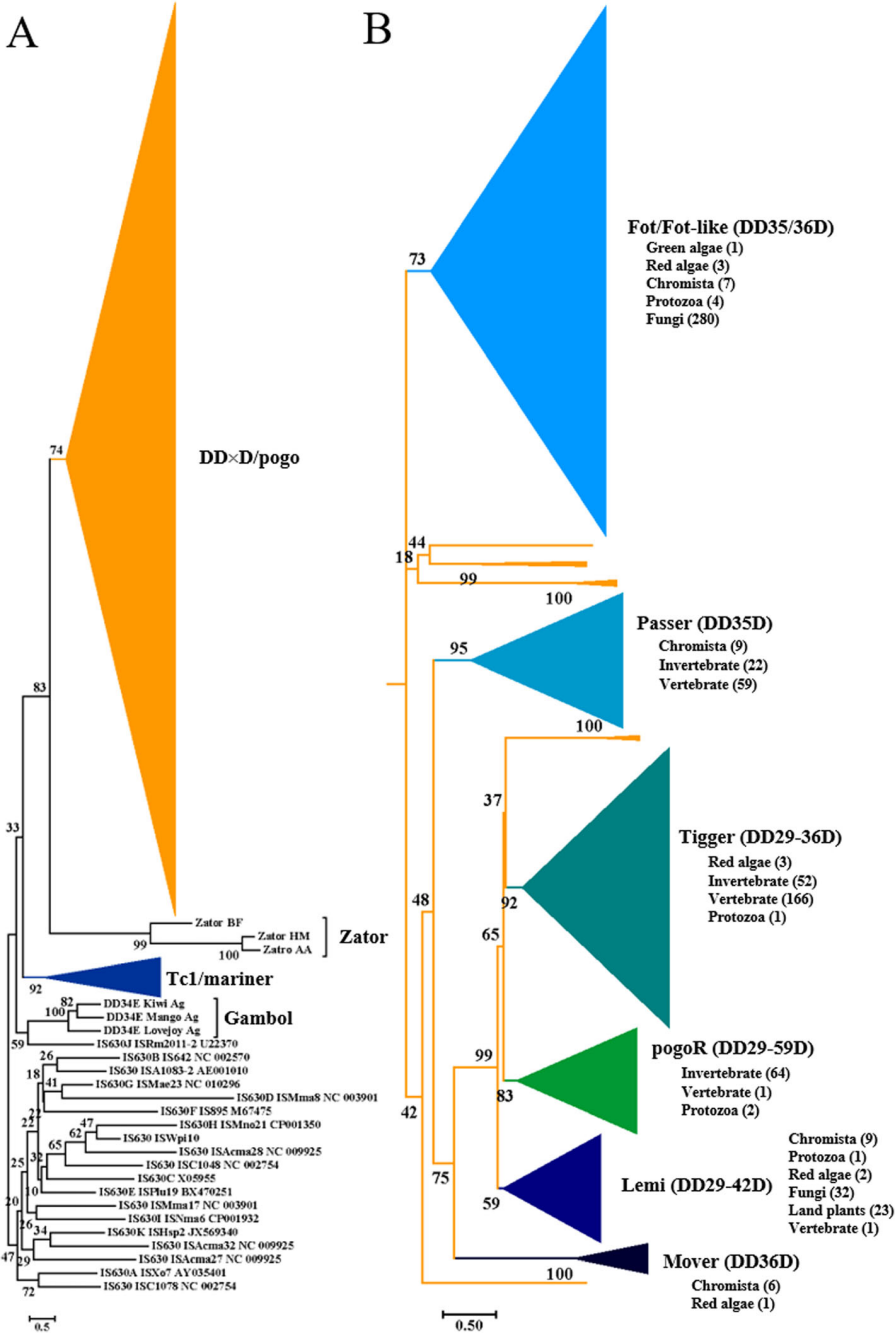
Phylogenetic tree of *pogo* transposases. **a** Unrooted phylogenetic tree of *pogo* transposases relative to *Zator*, *IS630*, DD34E/*Gambol*, and previously described *Tc1/mariner* families. The unrooted phylogenetic tree was inferred using the maximum likelihood method with the IQ-TREE program (see Additional file 2: Fig. S2 for an uncollapsed tree). Transposase sequences of DD36E/*IC*, DD35E/*TR*, DD37E/*TRT* are from the references [22, 23, 42]. GenBank access numbers of other reference elements are included in Additional file 2: Fig. S2. The number of species/organisms containing pogo elements for each *pogo* family is given in brackets for each lineage or group of eukaryotes

To define the phylogenetic position of *pogo* transposons, here we retrieved all bacteria *IS630* transposase sequences (121 sequences) containing DDE domains from ISfinder database [41], classified them into 11 clades by using the *IS256* transposase as outgroup (Additional file 1: Fig. S1). Then, 19 representative *IS630* transposase sequences including the 11 clades, *Tc1/mariner* transposase families identified previously [21–23, 26, 42–47], and *Zator* transposases, which were defined as a superfamily close to *ITm* group [28], were combined with all identified *pogo* transposases to infer a phylogenetic tree by using maximum likelihood methods with the IQ-TREE program [48]. The resulting tree shows that although *pogo*, *Tc1/mariner* (including DD34E/*Tc1*, DD35E/*TR*, DD36E/*IC*, DD34D/*mariner*, DD37D/*maT*, DD37E/*TRT*, DD37D, DD39D, and DD41D), *Zator*, and DD34E/*Gambol* transposases are sister clades, they formed four distinct highly supported monophyletic clades, with 74, 99, 92, and 100% bootstrap supports for *pogo*, *Zator*, *Tc1/mariner*, and DD34E/*Gambol* clades respectively (Fig. 1a and Additional file 2: Fig. S2). Therefore, we assume that *pogo*, *Tc1/mariner*, DD34E/*Gambol*, and *Zator* transposons may have evolved independently from *IS630* transposons and form separate superfamilies of eukaryotic DNA transposons. In order to investigate the origin of *pogo* transposons, we also conducted Blast searches against the bacteria genomes. However, we could not identify any other insertion sequences homologous to *pogo* transposons, beside *IS630* elements, indicating that *pogo* may still originate from the insertion sequences of *IS630* group. The *pogo* transposons were further classified into six main families (*Passer*, *Tigger*, *pogoR*, *Lemi*, *Mover*, and *Fot/Fot-like*). Four of them (*Fot/Fot-like* [49], *Tigger* [30] *pogoR* [29], and *Lemi* [37]) correspond to the known families reported previously, and have 73, 92, 83, and 59% bootstrap supports respectively, *pogoR* is the first *pogo* transposon identified in fly (*Drosophila melanogaster*) [29], while both *Passer* and *Mover* have been defined as new families with 95 and 100% bootstrap supports respectively (Fig. 1b). The main *pogo* superfamily also consists of a well-supported grouping including diverse minor clades (Fig. 1b and Additional file 2: Fig. S2).

### Extensive distribution of *pogo* transposons in eukaryotes

**Fig. 2:**
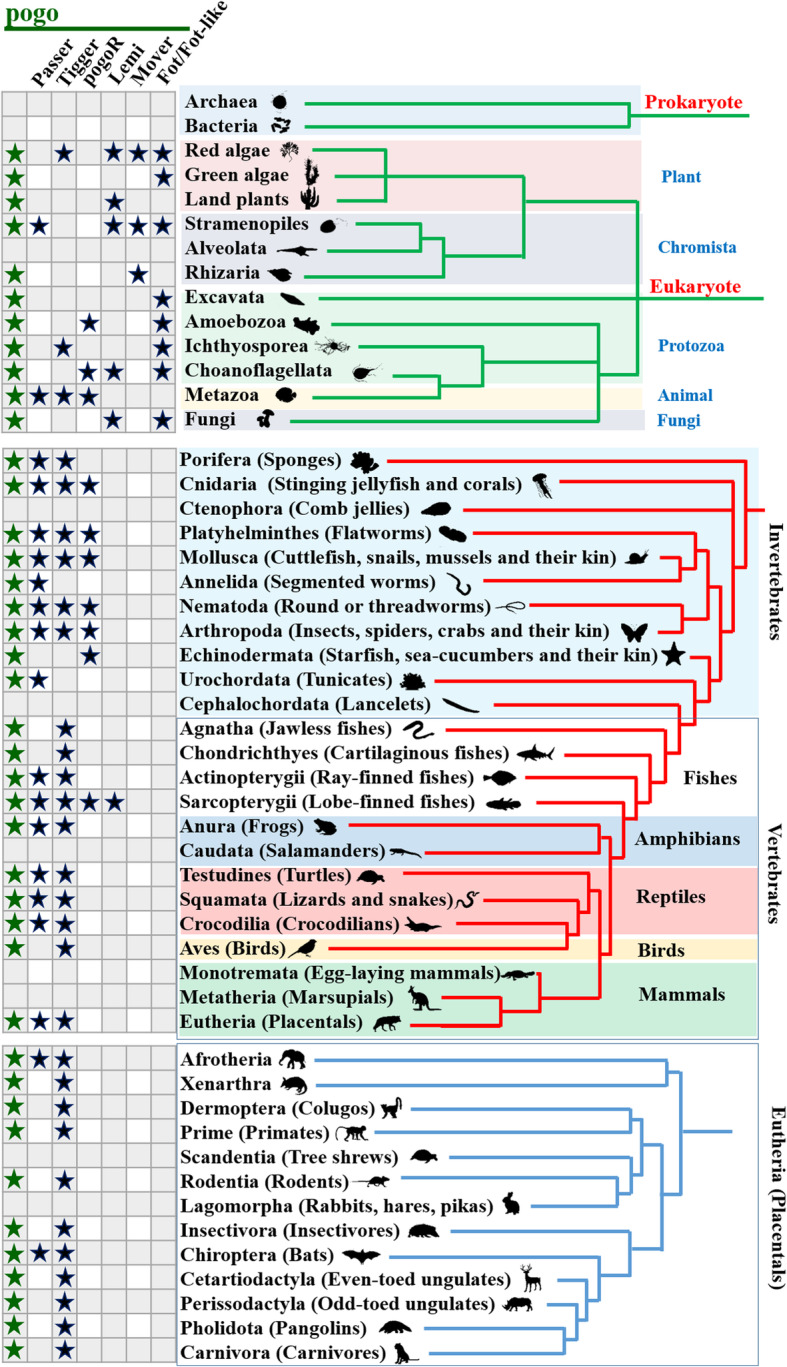
Distribution of *pogo* transposons. Symbols in green represent the distribution of all *pogo* transposons including *Passer*, *pogoR*, *Tigger*, *Lemi*, *Mover*, and the unclassified elements

The species in which *pogo* transposons were detected, their classification, structural characteristics, sequences and genome coordinates in each genome were listed in Additional file 3: Table S1. The *pogo* superfamily is absent from prokaryotes, but present in all kingdoms of eukaryotes, including plants (red algae, green algae, and land plants), *Chromista* (*Stramenopiles* and *Rhizaria*), protozoa (*Amoebozoa*, *Excavata*, *Choanoflagellata*, and *Ichthyosporea*), fungi, and animals. This superfamily is also distributed widely within the phyla and classes of invertebrates and vertebrates, only being absent from the *Ctenophora* and *Cephalochordata* of invertebrates and *Caudata* of vertebrates (Fig. 2).

Although three families (*pogoR*, *Passer*, and *Tigger*) are mainly present in the animal kingdom, only *Passer* and *Tigger* transposons displayed extensive distributions in vertebrates (Fig. 2). The *pogoR* transposons are mainly distributed within the invertebrates (64 species), including the *Cnidaria*, *Echinodermata*, *Platyhelminthes*, *Nematoda*, *Arthropoda*, and *Mollusca*, and only present in one species (*Latimeria chalumnae*/lobe-finned fish) of vertebrates (Figs. 1 and 2, and Additional file 3: Table S1). *Passer* forms a big family distributed in all detected phyla of invertebrates (22 species) except for the *Ctenophora* and *Cephalochordata*, most classes (ray-finned fish, lobe-finned fish, amphibians, reptiles, and mammals) of vertebrates (59 species), and nine species (*Stramenopiles*) of *Chromista*. Within mammals, *Passer* transposons are only found in two orders of eutherians (*Chiroptera* and *Afrotheria*) (Fig. 1 and Fig. 2, and Additional file 3: Table S1). *Tigger* also comprises a big family that was first reported in humans [30], and is distributed across most phyla of invertebrates (*Porifera*, *Cnidaria*, *Platyhelminthes*, *Nematoda*, *Arthropoda*, and *Mollusca*) (52 species) and all the classes of vertebrates (166 species), except for the *Caudata*. Some elements in three species of red algae are also defined as *Tigger* (Fig. 1 and Fig. 2, and Additional file 3: Table S1). Furthermore, *Tigger* transposons display extensive distribution within most orders of eutherian mammals (Fig. 2). We even identified *Tigger* transposons in most species of primates (Additional file 3: Table S1). However, the taxonomic distribution of these families including *Tigger* may be underestimated due to the exclusion of the truncated elements of ancient copies.

*Lemi* transposons are present in red algae (two species), land plants (23 species), *Chromista* (nine species), protozoa (one species), fungi (32 species), and animals (one species of lobe-finned fish), while *Mover* forms a small clade and displays a restricted distribution within red algae (one species) and *Chromista* (five species of *Stramenopiles* and one species of *Rhizaria*) (Fig. 1 and Fig. 2, and Additional file 3: Table S1). By contrast, *Fot/Fot-like* is a very large family, which also consists in multiple minor clades with varying bootstrap support (*Fot-like* elements) that share a sister-group relationship with a well-supported (100%) clade of the *Fot* family (Additional file 2: Fig. S2). *Fot* is distributed extensively in fungi (280 species), while *Fot-like* elements are distributed within the *Chromista* (seven species of *Stramenopiles*), protozoa (each species in each of the *Amoebozoa*, *Excavata*, *Choanoflagellata*, and *Ichthyosporea*), one species of green algae and three species of red algae (Figs. 1 and 2, and Additional file 3: Table S1).

### Wide occurrence of *pogo* transposons in fungi

**Fig. 3:**
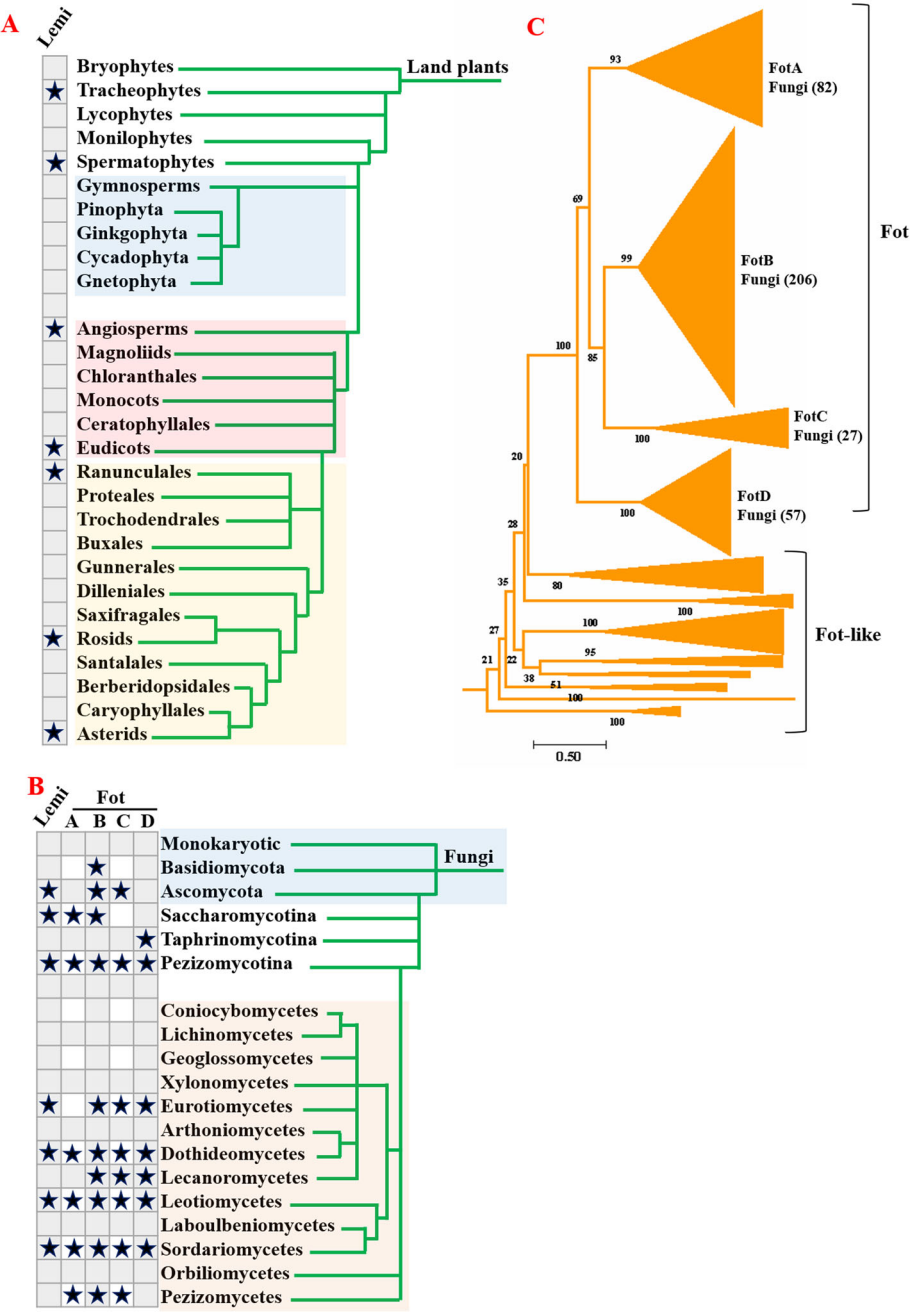
Distribution of *pogo* transposons in fungi and land plants. **a** Distribution of *Lemi* transposons in land plants. **b** Distribution of *Lemi* and *Fot* transposons in fungi. **c** The intra-family classification of *Fot* transposons. The number of species/organisms containing *Fot* elements for each clade is given in brackets

The *pogo* transposons were detected within most subgroups of plants, including red and green algae and land plants (Fig. 2). They did not undergo significant amplification among land plants, in which only one small clade of *pogo* transposons (named Lemi) was identified in 23 Eudicot species (one species in the Ranunculales, 16 Rosid species, and six Asterid species) (Fig. 1 and Fig. 3a, and Additional file 4: Fig. S3A and Additional file 3: Table S1). By contrast, wide distribution of *pogo* transposons was observed in fungi. One was defined as the *Lemi* family, distributed among two species of *Saccharomycotina*, and 30 species across four classes of *Pezizomycotina* (*Eurotiomycetes*, *Dothideomycetes*, *Leotiomycetes*, and *Sordariomycetes*; Fig. 1 and Fig. 3b, Additional file 3: Table S1 and Additional file 4: Fig. S3A). The other was defined as the *Fot* family, further classified into four distinctive clades (*FotA–D*), which displayed an extensive distribution in fungi; these were detected in 82, 206, 27, and 57 species, respectively, across six classes of *Pezizomycotina*/*Ascomycota* (*Eurotiomycetes*, *Dothideomycetes*, *Lecanoromycetes*, *Leotiomycetes*, *Sordariomycetes*, and *Pezizomycetes*) and two classes of *Basidiomycota* (Fig. 3b-c, Additional file 3: Table S1, and Additional file 4: Fig. S3B). In addition, the copy number of *Fot* elements in the genomes of different fungi species varies dramatically, from only one copy (> 90% of identity and > 1000 bp in length) to over hundred copies, but most of them have less than 200 copies (Additional file 3: Table S1).

### Significant structural variations of *pogo* transposons

**Fig. 4:**
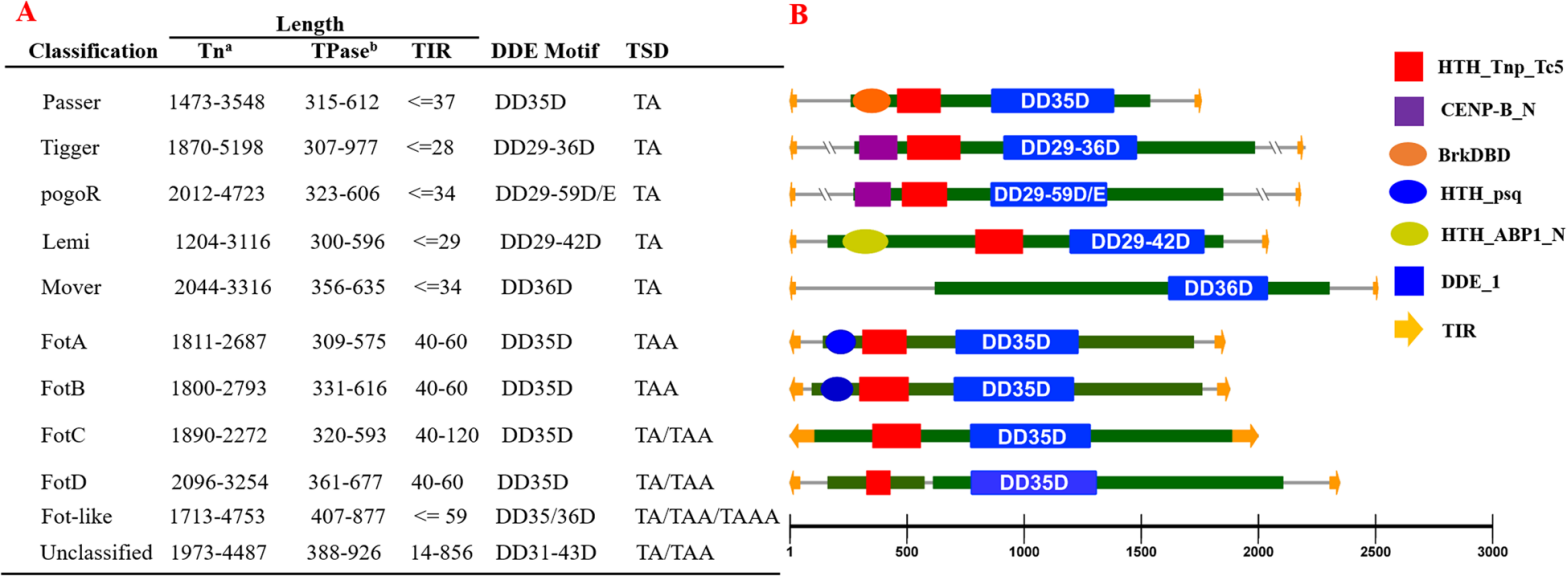
Structural organization of *pogo* transposons. **a** Summary of structural organization of *pogo* transposons. **b** Schematic of structural organization correspondence to each *pogo* transposon family/calde. The domain architecture of transposases was screened using hmmscan [50]; the names of DBD and DDE motifs have been designated according to the Pfam database [51]. ^a^The lengths of full transposons with detectable TIR and TSD sequences; ^b^The length of a putatively intact transposase (TPase) (> 300 aa) in a full-length transposon

The members of *pogo* superfamily have a total length range of 1.20–5.20 kilobases (kb), with one or two open reading frames (ORFs) encoding transposases of 300–977 amino acids (aa), flanked by TIRs and TSDs (Fig. 4). Significant structural variations were observed for *pogo* transposons. Two new types of TSDs (TAA and TAAA) were identified in *Fot* and *Fot-*like transposons, respectively, which differed from the classical 2 bp TA-flanked TSD of *Tc1/mariner* [52], while other *pogo* transposon families were still characterized by classical TA TSDs. Most *Fot* transposons (341 out of 385 elements) have TAA TSDs, while the rest *Fot* elements (54 out of 385 elements) have TA TSDs, which only present in *FotC* and *FotD* clades. Ten *Fot-like* elements have TAAA TSDs, while the other *Fot-like* transposons display TA (50 elements) or TAA (10 elements) TSDs. Four types of *pogo* transposon TIRs were identified: Type 1, ≤40 bp, found in most *pogo* transposon families; Type 2, 40–60 bp, identified in most *Fot* and *Fot*-like transposons; Type 3, a medium-length TIR (about 100 bp), which was mainly detected in *FotC* transposons; and Type 4, a very long TIR (392–856 bp) identified in some unclassified *pogo* transposons with a distribution restricted to insects (Fig. 4 and Additional file 3: Table S1). Different organizations of transposase domains, which were screened by using hmmscan [50], across the families and clades of *pogo* transposons were also observed. Significant variability was observed in the numbers of amino acids between the last two residues of the catalytic domain (DDE) of transposase for the families of *Tigger* (DD29–36D), *pogoR* (DD29–59D/E), and *Lemi* (DD29–42D). This number was highly conserved in most other *Tc1/mariner* families [26, 38, 42], while almost all *Passer* and *Fot/Fot-like* transposons were characterized by DD35D, and all *Mover* transposons by DD36D. Only four *Fot-like* transposons in *Ichthyosporea* are characterized by a DD36D domain. Furthermore, very large spacings (DD56D and DD59D) were observed for some *pogoR* transposons in *Amoebozoa* (Fig. 1 and Fig. 4, and Additional file 3: Table S1). Five types of DNA binding domain (DBD) motifs, designated CENP-B_N, HTH_ABP1_N, HTH_Tnp_Tc5, BrkDBD, and HTH_psq in the Pfam database [51], were identified in N-terminals of *pogo* transposases. *Fot* and *Fot*-like transposases harbored two types of motifs (HTH_psq and HTH_Tnp_Tc5), or a single motif of HTH_psq or HTH_Tnp_Tc5 in the DBD domain, while the *Passer* transposases harbored double DBD motifs of BrkDBD and HTH_Tnp_Tc5, or a single DBD motif of HTH_Tnp_Tc5. *Lemi* transposases are characterized by a single DBD motif (HTH_Tnp_Tc5), or double DBD motifs of HTH_ABP1_N and HTH_Tnp_Tc5. *pogoR* and *Tigger* transposases harbor double DBD motifs of CENP-B_N and HTH_Tnp_Tc5, or a single DBD motif of HTH_Tnp_Tc5. The DBD motif of *Mover* transposases, taxonomically restricted to red algae, and *Chromista*, was not detectable by hmmscan (Fig. 4 and Additional file 3: Table S1).

### Recurrent domestication events of *pogo* transposons in vertebrates

**Fig. 5:**
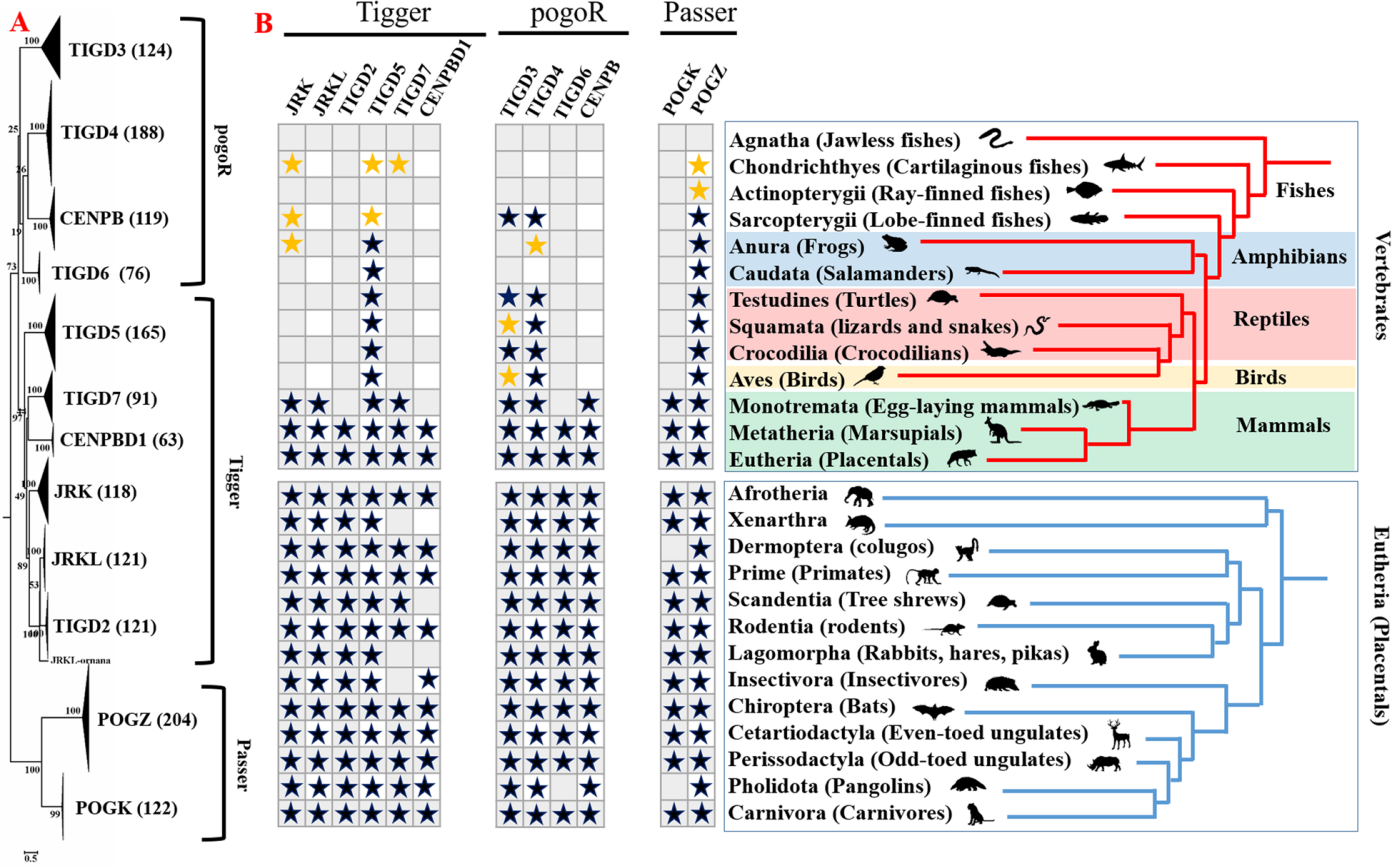
Classification and distribution of *pogo* transposase domesticated proteins. **a** Phylogenetic tree of *pogo* transposase domesticated proteins inferred using the maximum likelihood method (see Additional file 6: Fig. S4 for an uncollapsed tree). The other reference families of *Tc1/mariner* were included to infer an accurate tree, and the *DD35E/IS630* family was used as outgroup. The number of sequences in the *pogo* domesticated protein groups is given in brackets. The uncollapsed tree in Additional file 7: Fig. S5 includes the DDE domains from all *pogo* transposase sequences and domesticated proteins to deduce the origins of these proteins. **b** Distribution of *pogo* transposase domesticated proteins. Yellow stars represent pseudogenes, while black stars represent stationary domesticated genes

Based on an analysis of the RefSeq Representative Genome Database, we found that *pogo* transposons underwent recurrent domestication in vertebrates. Over 1500 protein sequences derived from *pogo* transposases were found, representing at least 12 well-annotated genes (Additional file 5: Table S2), including CENPBD1, JRK, JRKL, TIGD2–7, POGK and POGZ, beside the CENPB, which has been characterized previously [39]. The phylogenetic tree revealed that these protein sequences were derived from three families (*Passer*, *pogoR*, and *Tigger*) of *pogo* transposases, and can be classified into three groups: Group I, which includes four genes (TIGD3, TIGD4, TIGD6, and CENPB) derived from *pogoR* transposase; Group II, which includes five genes (CENPBD1, JRK, JRKL, TIGD2, TIGD5, and TIGD7) derived from *Tigger* transposase; and Group III, which includes POGK and POGZ derived from *Passer* transposase (Fig. 5a, Additional file 6: Fig. S4 and Additional file 7: Fig. S5). The continuous phylogenetic distribution of these genes, coupled with high sequence identity (> 74%) and low nonsynonymous to synonymous substitutions (Ka/Ks) ratios (< 1) (Z-test, *P* < 0.05) (Fig. 5b and Table 1), which provides a measure of selection acting to maintain amino acid sequence [53], strongly suggests that they evolved under strong purifying (negative) selection, and tend to represent stationary domesticated genes. In addition, we also identified many TIGD1s and TIGD1-like sequences (TIGD1Ls), which are homologous to *Tigger* transposases; however, all TIGD1s and most TIGD1Ls are present as multiple copies. The TIGD1Ls were grouped into several small clades displaying low sequence identities and very narrow distribution among taxa (data not shown), indicating that they are akin to pseudogenes, and therefore excluded from this analysis.

**Table 1:** Features of *pogo* transposase domesticated proteins identified in vertebrate

Group	Gene	Protein length (aa)	Sequence identity (Species number)	Ka / Ks ratio (Species number / Z-test)	Conserved domains			
					DBD domain	DDE domain	Catalytic triad	Additional functional domains
***Tigger*-derived**	**TIGD2**	**~ 525**	**91.62 ± 0.05 (*n*** **= 121)**	**0.093 ± 0.029 (*n*** **= 120 /*P < 0.05*)**	**CENP-B_N/HTH_Tnp_Tc5**	**DDE_1**	**DD34S**	
	**JRKL**	**~ 524**	**92.93 ± 0.08 (n = 121)**	**0.055 ± 0.022 (*n*** **= 117 /*P < 0.05*)**	**CENP-B_N/HTH_Tnp_Tc5**	**DDE_1**	**DD34D/N**	
	**JRK**	**~ 530–570**	**74.60 ± 0.10 (*n*** **= 118)**	**0.207 ± 0.073 (*n*** **= 114 /*P < 0.05*)**	**CENP-B_N/HTH_Tnp_Tc5**	**DDE_1**	**DD32D/E**	
	**TIGD5**	**~ 530–670**	**74.13 ± 0.10 (*n*** **= 165)**	**0.176 ± 0.099 (*n*** **= 161 / P < 0.05)**	**CENP-B_N/HTH_Tnp_Tc5**	**DDE_1**	**?**	
	**TIGD7**	**~ 550**	**87.45 ± 0.10 (*n*** **= 91)**	**0.171 ± 0.066 (*n*** **= 87 /*P < 0.05*)**	**CENP-B_N/HTH_Tnp_Tc5**	**DDE_1**	**DD34N**	
	**CENPBD1**	**~ 540**	**79.50 ± 0.36 (*n*** **= 63)**	**0.177 ± 0.098 (*n*** **= 62 /*P < 0.05*)**	**CENP-B_N/HTH_Tnp_Tc5**	**DDE_1**	**DD32G**	
***pogoR*-derived**	**CENPB**	**~ 600**	**89.80 ± 0.09 (*n*** **= 119)**	**0.090 ± 0.062 (*n*** **= 115 /*P < 0.05*)**	**CENP-B_N/HTH_Tnp_Tc5**	**DDE_1**	**?**	**CENP-B_dimeris**
	**TIGD6**	**~ 520**	**87.08 ± 0.15 (*n*** **= 76)**	**0.207 ± 0.140 (*n*** **= 76 /*P < 0.05*)**	**CENP-B_N/HTH_Tnp_Tc5**	**DDE_1**	**DD30N**	
	**TIGD3**	**~ 470**	**80.63 ± 0.11 (*n*** **= 124)**	**0.195 ± 0.069 (*n*** **= 123 /*P < 0.05*)**	**CENP-B_N/HTH_Tnp_Tc5**	**DDE_1**	**?**	
	**TIGD4**	**~ 510**	**77.74 ± 0.08 (*n*** **= 188)**	**0.142 ± 0.050 (*n*** **= 186 /*P < 0.05*)**	**CENP-B_N/HTH_Tnp_Tc5**	**DDE_1**	**?**	
***Passer*-derived**	**POGK**	**~ 610**	**90.75 ± 0.08 (*n*** **= 122)**	**0.062 ± 0.043 (*n*** **= 117 /*P < 0.05*)**	**BrkDBD/HTH_Tnp_Tc5**	**DDE_1**	**DN35D**	**KRAB**
	**POGZ**	**~ 1200–1410**	**83.63 ± 0.08 (*n*** **= 204)**	**0.068 ± 0.048 (*n*** **= 198 /*P < 0.05*)**	**HTH_Tnp_Tc5**	**DDE_1**	**DD35D**	**ZNF**

Based on the phylogenetic analysis and the Ka/Ks ratio analysis, the stationary domesticated genes were confirmed, and their taxonomic distribution was summarized in Fig. 5a and b. Together, these data revealed four genes (TIGD3, TIGD4, TIGD5, and POGZ) tend to represent ancient domestication events in vertebrates, while eight (JRK, JRKL, TIGD2, CENPB, CENPBD1, TIGD6, TIGD7, and POGK) appear to be present as recent domestication events in mammals (Fig. 5b). TIGD3 and TIGD4 display continuous distributions in mammals, but have a more uneven phyletic distribution in non-mammalian vertebrates. TIGD3, TIGD4, and POGZ were likely recruited to the *Sarcopterygii*/lobe-finned fish superclass, prior to the split of *Amniota* and *Amphibia*, but TIGD3 was subsequently lost from the *Anura*, *Caudata*, *Squamata*, and *Aves*, while TIGD4 was lost from the *Anura* and *Caudata* (Fig. 5b). Pseudogenes of TIGD3 were detected in *Squamata* and *Aves*, those of TIGD4 were detected in *Anura,* which are phylogenetically close to the stationary domesticated genes, but forming a distinct clade and displaying low sequence identity within clades (Additional file 5: Table S2 and Additional file 7: Fig. S5). TIGD5 has emerged in *Amniota* and *Amphibia*, and both TIGD5 and POGZ display continuous distributions and seem to have been maintained in most lineages of vertebrates after domestication. CENPB, JRK, JRKL, TIGD7, and POGK might have originated in the egg-laying mammals (*Monotremata*), prior to the divergence of the marsupials and eutherian (“placental”) groups, while TIGD2, TIGD6, and CENPBD1 seem to have emerged in the *Theria*. All these genes display continuous distributions in mammals except for CENPBD1, which is absent from the *Lagomorpha*, *Scandentia*, and *Xenarthra*, and missing from most species of primates and rodents, but has continuous distribution in the *Laurasiatheria* (*Chiroptera*, *Cetartiodactyla*, *Perissodactyla*, *Pholidota*, and *Carnivora*; Fig. 5b and Additional file 5: Table S2), suggesting that it might be a very recent domestication event in mammals. Pseudogenes of these genes were also detected: JRK in cartilaginous fish and lobe-finned fish, and *Anura*; TIGD7 in cartilaginous fish; TIGD5 in cartilaginous and lobe-finned fish; and POGZ in cartilaginous and ray-finned fish (Fig. 5b, Additional file 5: Table S2 and Additional file 7: Fig. S5). In addition, the phylogenetic tree also suggests that most genes arose by independent domestication events from different sources of *pogo* transposases. However, JRKL and TIGD2 appeared to emerge from a common transposase ancestor, and JRKL in the *Monotremata* seems to be the common ancestral gene of TIGD2 and JRKL, providing evidence for a co-domestication event of *pogo* transposons in vertebrates (Fig. 5b, Additional file 6: Fig. S4 and Additional file 7: Fig. S5).

### Structural conservation of *pogo* transposase domesticated genes

**Fig. 6:**
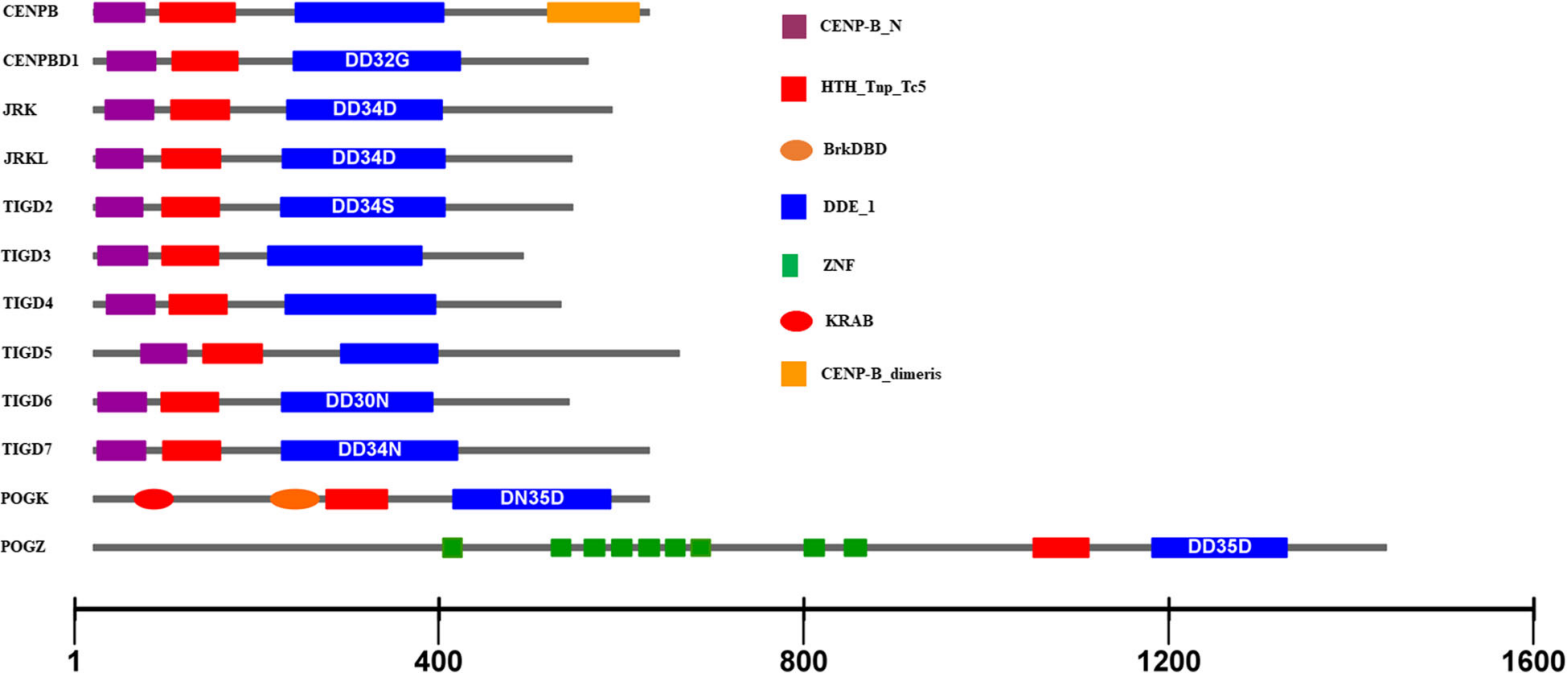
Structural organization of *pogo* transposase domesticated proteins. The domain architecture of domesticated proteins was screened using hmmscan [50], and the names of the identified motifs were designated according to the Pfam database [51]

Examination of the domain architecture of these domesticated proteins compared with *pogo* transposases revealed that most domesticated genes are derived from the complete transposase genes and show the same DBD and DDE domains found in *pogo* transposases; three of them (CENPB, POGK, and POGZ) are chimeric genes emerging from the fusion of entire transposase genes with additional functional domains (Fig. 6, Additional file 5: Table S2 and Additional file 8: Fig. S6). CENPB obtained an additional domain of dimerization in the C-terminal region, while POGK and POGZ obtained a Kruppel-associated box (KRAB) and zinc finger (ZNF) domain near the N-terminus, respectively (Fig. 6, Additional file 5: Table S2 and Additional file 8: Fig. S6A-6C). Seven to nine ZNF finger motifs scattered in the N-terminal of POGZs were identified (Additional file 8: Fig. S6C), which are now recognized to bind DNA, RNA, protein, and/or lipid substrates [54]. The DDE domain has been fully retained in all domesticated genes. The triad signatures of the DDE domain are well conserved in the JRK, JRKL, and POGZ sequences, and partially conserved in CENPBD1, TIGD2, TIGD6, TIGD7, and POGK, but are not recognizable in CENPB, TIGD3, TIGD4, and TIGD5; Most domesticated genes harbor two type DBD motifs represented by CENP-B_N and HTH_Tnp_Tc5 in the N-terminal: POGKs are by BrkDBD and HTH_Tnp_Tc5 DBD motifs, while POGZs harbor only one DBD motif (HTH_Tnp_Tc5; Fig. 6, Additional file 5: Table S2 and Additional file 8: Fig. S6).

## Discussion

We report a comprehensive analysis of the evolutionary history of *pogo* transposons in eukaryotes and of their repeated domestication in vertebrates. Many domesticated genes derived from DNA transposases have been characterized previously [11], with only SETMAR [13] and CENPB [39] known to derive from the *Tc1/mariner* superfamily. JRK, JRKL, and TIGD1–7 are also known as domesticated from *pogo* transposases [39], however, their structure organization, origins and taxonomic distribution are largely unknown. Our results first point to the common domestication events of *pogo* transposons—as a superfamily of *ITm* transposons—in vertebrates, where 12 well-annotated genes (CENPBD1, JRK, JRKL, POGK, POGZ, and TIGD2–7) beyond CENPB [39] were predicted to have evolved from this family. They were domesticated at different times during the evolution of vertebrates, with some very ancient domestication events leading to TIGD3, TIGD4, TIGD5, and POGZ. These genes first emerged in lobe-finned fish or *Amphibia*, similar to RAG1 [14] and PBGD5 [55]. More recent domestication events of CENPB, JRK, JRKL, POGK, and TIGD7 occurred in the ancestor of Mammalia, prior to the divergence of *Theria* and *Monotremata*, and TIGD2 and TIGD6 emerged after the split of the marsupials and eutheria, while the very recent domestication of CENPBD1 is present only in some mammalian lineages. Here, we clearly defined the putative origins of these domesticated genes for the first time. Our data suggests that only TIGD2, TIGD5, and TIGD7 originated from *Tigger* transposases, while TIGD3, TIGD4, and TIGD6 are derived from *pogoR* transposases. Furthermore, the phylogenetic tree we constructed revealed that most genes have emerged from different transposons and represent independent domestication events. However, our analysis also suggests that TIGD2 and JRKL seem to have originated from the same *Tigger* transposon, and might represent a co-domestication event similar to the one found in bony vertebrates (HARBI1 and NAIF1) [56] and *Drosophila* (DPLG7 and DPMG7) [57]. The functions of these genes seem to be related to DNA binding, and both POGZ and CENPB are believed to act in modulating chromatin structure [40, 58]. It has been demonstrated that disruptions of POGZ are associated with autism [59–61], while inactivation of JRK in mice result in epileptic seizures [62], and JRK was found to be overexpressed in cancers [63].

Our study provides a global overview of the evolutionary relationships among *pogo*, *Tc1/mariner*, *Gambol*, and *Zator* transposons and refines the classification of the *ITm* group. *Zator* has been found to derive from *TP36* insertion sequences in bacteria [28], and suggested to be a separate superfamily being distantly related to the *Tc1/mariner* and bacterial *IS630* elements. However, both of *pogo* and *Gambol* once were classified as the families of *Tc1/mariner* transposons [25, 26, 47]. *Gambol* was identified in African malaria mosquito, and is characterized by a typical DD34E catalytic triad and TA TSDs. However, it was found to form a distinct group separated from DD34E/*Tc1* according to previous phylogenetic analyses [47]. Here, our phylogenetic analysis including more *IS630* representative sequences from the ISfinder database demonstrated that *pogo*, *Gambol*, and *Tc1/mariner* form well-supported monophyletic clades. We thus conclude that they are separate superfamilies that may have originated from different clades of bacterial *IS630* TEs and evolve independently, like *Zator*.

Our findings also suggest that *pogo* transposons might display the widest taxonomic distribution compared with the other *Tc1/mariner* superfamilies, as well as other superfamilies of DNA transposons [18]. The *DD34D/mariner* and *DD34E/Tc1* families have been examined in detail. *DD34D/mariner* was once regarded as the most widely distributed family of transposons, represented in diverse taxa such as fungi, ciliates, rotifers, insects, nematodes, plants, fish, and mammals [18]. However, we found that the *pogo* transposons are even more widely distributed in nature, with an expansion spanning across all kingdoms of eukaryotes. In addition, the taxonomic distribution of *pogo* transposons were underestimated since the ancient elements with truncated TIRs were excluded from our analysis. Our analysis also indicates that this superfamily has undergone a massive amplification in fungi with a wide taxonomic distribution, and is widespread in animals, where *pogo* transposons invaded almost all phyla of invertebrates and most classes of vertebrates, suggesting that the *pogo* transposons have played important roles in shaping the evolution of fungal and animal genomes. However, they did not accumulate significantly in land plants—being found in only 23 species—indicating a restricted influence of this superfamily on plant genome evolution.

We discovered that the *pogo* superfamily displays an unexpected level of diversity at the family and clade levels with significant variations in structural organization. Compared with other DNA transposons, *pogo* might represent the highest such diversity, with at least six distinct families defined (*Tigger*, *pogoR*, *Lemi*, *Mover*, *Passer*, and *Fot/Fot-like*). Furthermore, some families also display intra-group diversity and contain distinctive multiple clades, such as *Fot*/*Fot-like*, where at least four distinct clades (*FotA-D*) with well-supported bootstraps (> = 93%) were identified. Compared with the classic structures of *Tc1/mariner* transposons [16, 25], we found that *pogo* transposons show significant structural variations, including the transposon hallmarks of TSD and TIR sequences and the transposase domains DBD and DDE. Two new types of TSD (TAAA and TAA) have now been identified in *pogo* transposons in addition to the general TSD type (TA) of the *Tc1/mariner* superfamily. Significant variability (DD29–56D) of the number of amino acids between the last two residues of the triad signatures of the DDE domain was observed across different families, or between different clades of the same family, which is unique compared with most other families of *Tc1/mariner*.

## Conclusions

This is the first report to systematically revealing the evolutionary profiles of the *pogo* transposons, which was defined as a new superfamily of the *ITm* group and displays a high family diversity and very wide taxonomic distribution in nature, with a massive amplification in fungi and animals, but narrow distribution in land plants. Furthermore, we also provided evidence to support that *pogo* superfamily has been domesticated repeatedly in vertebrates, over 10 functional genes were deduced as originating from this superfamily. Ten of these originate from different sources of *pogo* transposases and represent independent domestication events, while two of them seem to have been co-domesticated. This study expands our understanding of the evolution of *ITm* transposons, and these data suggest that the *pogo* superfamily contributes significantly to diversifying and shaping the genomes of fungi and animals, as well as functional genes in vertebrates.

## Materials and methods

### Transposons mining

To determine the distributions of *pogo* transposons, 2612 sequences of *Tc1/mariner* transposons were downloaded from the RepBase (20181026) database [64] and combined with six sequences of pogo-like transposons from different teleost species, including cod (*Gadus morhua*), medaka (*Oryzias latipes*), stickleback (*Gasterosteus aculeatus*), tetraodon (*Tetraodon nigroviridis*), tilapia (*Oreochromis niloticus*), and zebrafish (*Danio rerio*), which were identified in our previous studies [9, 38], to generate 964 transposase sequences (> 300 aa); 302 sequences were identified as *pogo* transposases based on the phylogenetic analysis according to the references [9, 38]. Then, these *pogo* transposase sequences were used as queries to search against the available organism genomes, including prokaryotes (bacteria and archaea) and eukaryota, which comprise plants (red algae, green algae, and land plants), *Chromista* (*Stramenopiles*, *Alveolates*, and *Rhizaria*), protozoa (*Amoebozoa*, *Excavata*, *Ichthyosporea*, and *Choanoflagellata*), fungi, and animals, at the database of the National Center for Biotechnology Information (NCBI) by using TBlastN with a cutoff value of 1^e–100^. The new sequences identified were then used as queries to identify more elements. The top 10 non-overlapping hits were extracted along with 2 kb of flanking sequences, and aligned using the MAFFT program [65] to identify the transposon boundaries manually. Elements with two detectable TIRs and TSDs of DNA transposons, or elements coding for transposases of at least 300 aa with one TIR and TSD, are referred to as transposons, the truncated elements with only one TIR coding for transposases of less than 300 aa or undetectable TIRs were discarded, which may be ancient invasion copies. Then, these representative sequences were subjected to BLAST analysis of each host genome to estimate copy numbers. All BLAST hits > 1000 bp in size and > 80% identity were used to calculate copy numbers. In addition, the transposons with very few copies (< 3) in genomes, which may be false positives due to the sequence contamination, the flanking sequences of these transposons were further mapped to the host genome or the closely related species genomes, the un-mapping transposons were excluded for the analysis.

### Domesticated gene mining

The domesticated genes of these transposons were identified in the vertebrate species only with the Reference and Representative genomes deposited in RefSeq Representative Genome Database of NCBI, where the genomes in this database were well assembled and are among the best quality genomes available at NCBI. The domesticated genes were identified using the representative *pogo* transposases from different subfamilies as queries search against the NCBI genome databases available by using TBlastN with a cutoff value of 1^e–100^. Here, to discriminate between transposons sequences from domesticated genes, TBlastN was used to align each sequence with 2 kb flanking sequences on the host genome to detect potential TIR and TSD sequences. When TIRs and TSDs were found on both sides or one side, the sequence was considered to be a transposon, while sequences flanked by no TIR or TSD sequences were considered to be putatively domesticated genes. The structure of each domesticated gene sequence obtained using the TblastN program was predicted initially using GENSCAN (http://hollywood.mit.edu/GENSCAN.html) and refined by alignment with orthologous genes. Sequences used as vectors were removed, and in case of isoform proteins, only one sequence was selected. The remaining sequences were then submitted for classification and phylogenetic analysis. The average sequence identity of proteins was estimated by the multiple sequence alignment program (emma) embedded in EMBOSS (http://www.bioinformatics.nl/emboss-explorer/). It is not easy to distinguish transposons from transposon-derived genes in those genomes where large amounts of related and recently active transposons are found; therefore, we applied a stringent standard to filter out ambiguous domesticated genes. Gene clades with a low average sequence identity of proteins (< 70%), very narrow taxonomic distribution (fewer than five species), or multiple copies (> 3) in genomes were excluded from the domestication analysis.

### Domain architecture and phylogenetic analysis

The protein domains were identified using hidden Markov Models with the online hmmscan web server (https://www.ebi.ac.uk/Tools/hmmer/search/hmmscan) [50]. The ZFN sequence was predicted using an online web server (http://zf.princeton.edu/logoMain.php). To define the phylogenetic position of *pogo* transposons accurately, all bacteria *IS630* transposase sequences were retrieved from ISfinder database [41] and the DDE domains were extracted by using hmmalign program in HMMER (v3.3, http://hmmer.org/). Then, they (121 sequences) were aligned with MAFFT program [66] and submitted for classification by using the maximum likelihood method within IQ-TREE (v. 1.6.1) [48]. The bacteria *IS256* transposase was used as outgroup. Then, the representative sequences of *IS630* transposases from each clade and other unclassified sequences, *Tc1/mariner* known families transposases [21–23, 26, 42–47], and *Zatror* transposases [28] were jointed with the *pogo* transposases to infer the phylogenetic tree based on the multiple amino acid alignment of the conserved DDE domain by using the maximum likelihood method within IQ-TREE [48]. The best-fit model was selected by ModelFinder embedded in IQ-TREE [48], and the reliability of maximum likelihood trees was estimated by using the ultrafast bootstrap approach with 1000 replicates. The evolutionary histories of the domesticated proteins of *DD×D* transposases were inferred based on the alignments of DDE domains by using the IQ-TREE program as well [48], but the *IS630* family was used as an outgroup.

### Codon substitution pattern and statistical analysis

Coding sequences for domesticated genes of *pogo* transposases in vertebrates were aligned using ClustalW embedded in MEGA 7.0.26 [67], and the number of nonsynonymous substitutions per nonsynonymous site (Ka) and the number of synonymous substitutions per synonymous site (Ks) were estimated using the Nei–Gojobori method. The codon-based tests of selection analyses were conducted in MEGA with a Z test by calculating the substitution ratio of Ka/Ks [53]. Then, the Ka/Ks ratios were calculated to assess selection pressure using Z tests. The variance of the difference was computed using the bootstrap method (100 replicates). Orthologous sequences with a Ka/Ks value of < 1 (Z-test, *P* < 0.05) were defined as having been under purifying selection.

## Supplementary information

**Additional file 1: Fig. S1.***IS630* transposase classification. The phylogenetic tree was inferred using the maximum likelihood method with the IQ-Tree program, as described in the Materials and Methods. *IS256* transposase was used as an outgroup.

**Additional file 2: Fig. S2.** Uncollapsed phylogenetic tree of *pogo* transposases. The phylogenetic tree was inferred using the maximum likelihood method with the IQ-Tree program, as described in the Materials and Methods.

**Additional file 3: Table S1.** Distribution of *pogo* transposons. List of all species/organism containing *pogo* transposon. For each species, the following information is provided: classification information of species, transposon Name ID, structural characteristics of representative transposon, including copy number (Blast hits > 1000 bp and identity > 80%), transposon (Tn) length, transposase (Tpase) length (> 300 aa), TIR length, TIR end motif, TIR sequence, TSD, domains of transposase, and triad signature of the DDE domain, and genome coordinate and sequence of representative transposon.

**Additional file 4: Fig. S3.** The intra-group classification and distributions of *Lemi* and *Fot* transposons in fungi and land plants. (A) Subphylogenetic tree of *Lemi* transposases constructed using the maximum likelihood method. (B) Subphylogenetic tree of *Fot* transposases constructed using the maximum likelihood method. The number of species/organisms containing *Lemi* and *Fot* elements for each clade is given in brackets.

**Additional file 5: Table S2.** Distribution of *pogo* transposon domesticated proteins.

**Additional file 6: Fig. S4.** Uncollapsed phylogenetic tree of *pogo* transposases domesticated proteins. The tree was inferred using the maximum likelihood method with the IQ-Tree program, as described in the Materials and Methods. The *DD35E/IS630* family was used as an outgroup.

**Additional file 7: Fig. S5.** Uncollapsed phylogenetic tree of *pogo* transposons domesticated proteins including *pogo* transposases. The tree was inferred using the maximum likelihood method in the IQ-Tree program, as described in the Materials and Methods. The *DD35E/IS630* family was used as an outgroup. The pseudogenes (PS) of JRK, TIGD3, TIGD5, TIGD7, and POGZ are labeled as JRK PS, TIGD3 PS, TIGD5 PS, TIGD7 PS, and POGZ PS, respectively.

**Additional file 8: Fig. S6.** Alignment of *pogo* transposase domesticated proteins.

## Data Availability

All data needed to evaluate the conclusions in this paper are present either in the main text or the Supporting information.
