# Supplementary figures and images for: Evolution of pogo, a separate superfamily of IS630-Tc1-mariner transposons, revealing recurrent domestication events in vertebrates
Source: Mob DNA. 2020 Jul 22;11:25. doi: 10.1186/s13100-020-00220-0 (PMC7386202; doi:10.1186/s13100-020-00220-0)

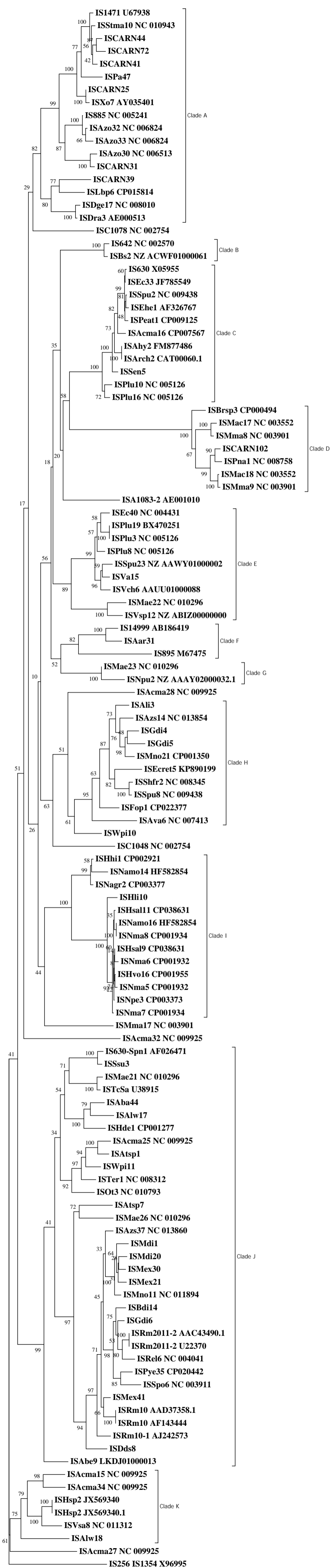

Supplement: Supplementary file 1 — Additional file 1: Fig. S1.IS630 transposase classification. The phylogenetic tree was inferred using the maximum likelihood method with the IQ-Tree program, as described in the Materials and Methods. IS256 transposase was used as an outgroup. [file 13100_2020_220_MOESM1_ESM.pdf]

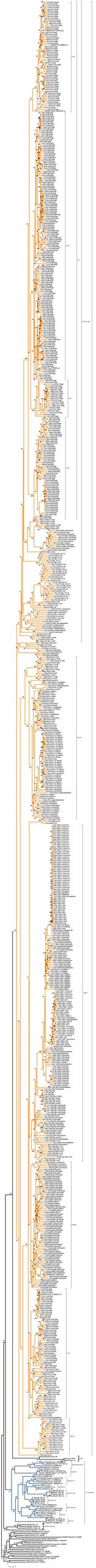

Supplement: Supplementary file 2 — Additional file 2: Fig. S2. Uncollapsed phylogenetic tree of pogo transposases. The phylogenetic tree was inferred using the maximum likelihood method with the IQ-Tree program, as described in the Materials and Methods. [file 13100_2020_220_MOESM2_ESM.pdf]

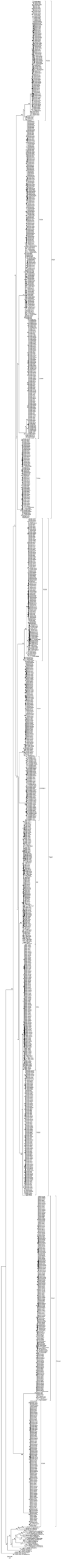

Supplement: Supplementary file 6 — Additional file 6: Fig. S4. Uncollapsed phylogenetic tree of pogo transposases domesticated proteins. The tree was inferred using the maximum likelihood method with the IQ-Tree program, as described in the Materials and Methods. The DD35E/IS630 family was used as an outgroup. [file 13100_2020_220_MOESM6_ESM.pdf]
