# Supplementary material for: Evolution of pogo, a separate superfamily of IS630-Tc1-mariner transposons, revealing recurrent domestication events in vertebrates
Source: Mob DNA. 2020 Jul 22;11:25. doi: 10.1186/s13100-020-00220-0 (PMC7386202; doi:10.1186/s13100-020-00220-0)
Supplement: Supplementary file 4 — Additional file 4: Fig. S3. The intra-group classification and distributions of Lemi and Fot transposons in fungi and land plants. (A) Subphylogenetic tree of Lemi transposases constructed using the maximum likelihood method. (B) Subphylogenetic tree of Fot transposases constructed using the maximum likelihood method. The number of species/organisms containing Lemi and Fot elements for each clade is given in brackets. [file 13100_2020_220_MOESM4_ESM.pdf]

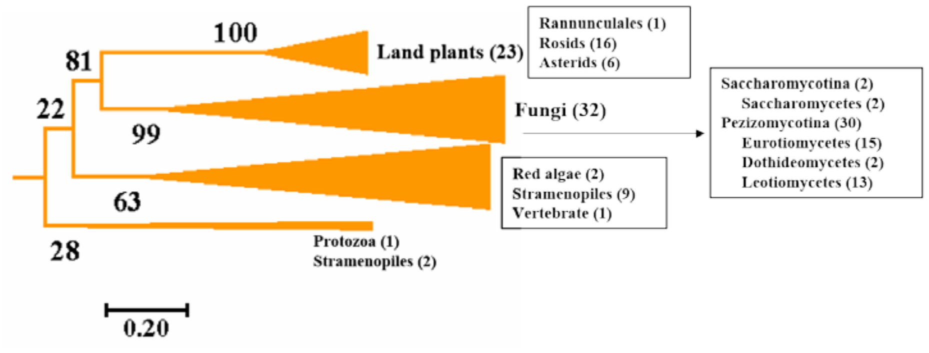

A. Subphylogenetic tree of Lemi

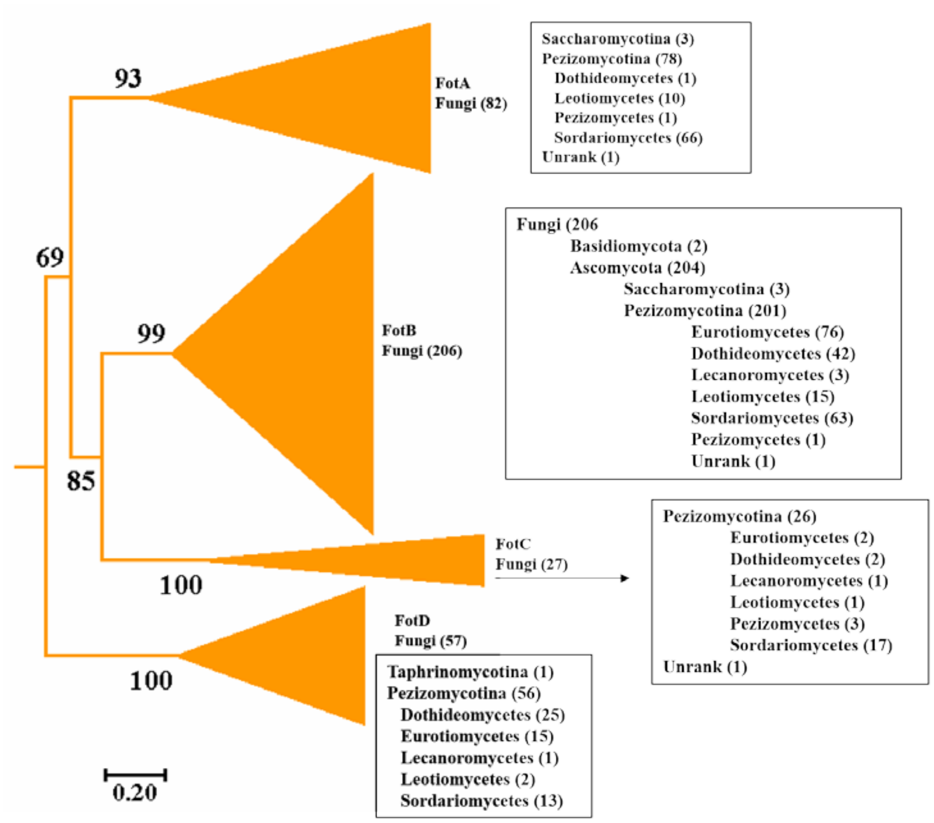

B. Subphylogenetic tree of Fot

Figure S3. The intra-group classification and distributions of *Lemi* and *Fot* transposons in fungi and land plants. (A) Subphylogenetic tree of *Lemi* transposases constructed using the maximum likelihood method. (B) Subphylogenetic tree of *Fot* transposases constructed using the maximum likelihood method. The number of species/organisms containing *Lemi* and *Fot* elements for each clade is given in brackets.
