# Supplementary material for: Evolution of pogo, a separate superfamily of IS630-Tc1-mariner transposons, revealing recurrent domestication events in vertebrates
Source: Mob DNA. 2020 Jul 22;11:25. doi: 10.1186/s13100-020-00220-0 (PMC7386202; doi:10.1186/s13100-020-00220-0)

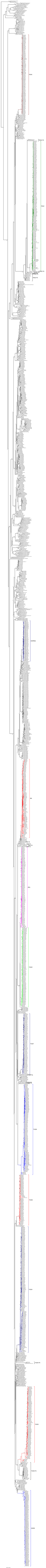

Supplement: Supplementary file 7 — Additional file 7: Fig. S5. Uncollapsed phylogenetic tree of pogo transposons domesticated proteins including pogo transposases. The tree was inferred using the maximum likelihood method in the IQ-Tree program, as described in the Materials and Methods. The DD35E/IS630 family was used as an outgroup. The pseudogenes (PS) of JRK, TIGD3, TIGD5, TIGD7, and POGZ are labeled as JRK PS, TIGD3 PS, TIGD5 PS, TIGD7 PS, and POGZ PS, respectively. [file 13100_2020_220_MOESM7_ESM.pdf]
