# Supplementary material for: Evolution of pogo, a separate superfamily of IS630-Tc1-mariner transposons, revealing recurrent domestication events in vertebrates
Source: Mob DNA. 2020 Jul 22;11:25. doi: 10.1186/s13100-020-00220-0 (PMC7386202; doi:10.1186/s13100-020-00220-0)
Supplement: Supplementary file 8 — Additional file 8: Fig. S6. Alignment of pogo transposase domesticated proteins. [file 13100_2020_220_MOESM8_ESM.pdf]

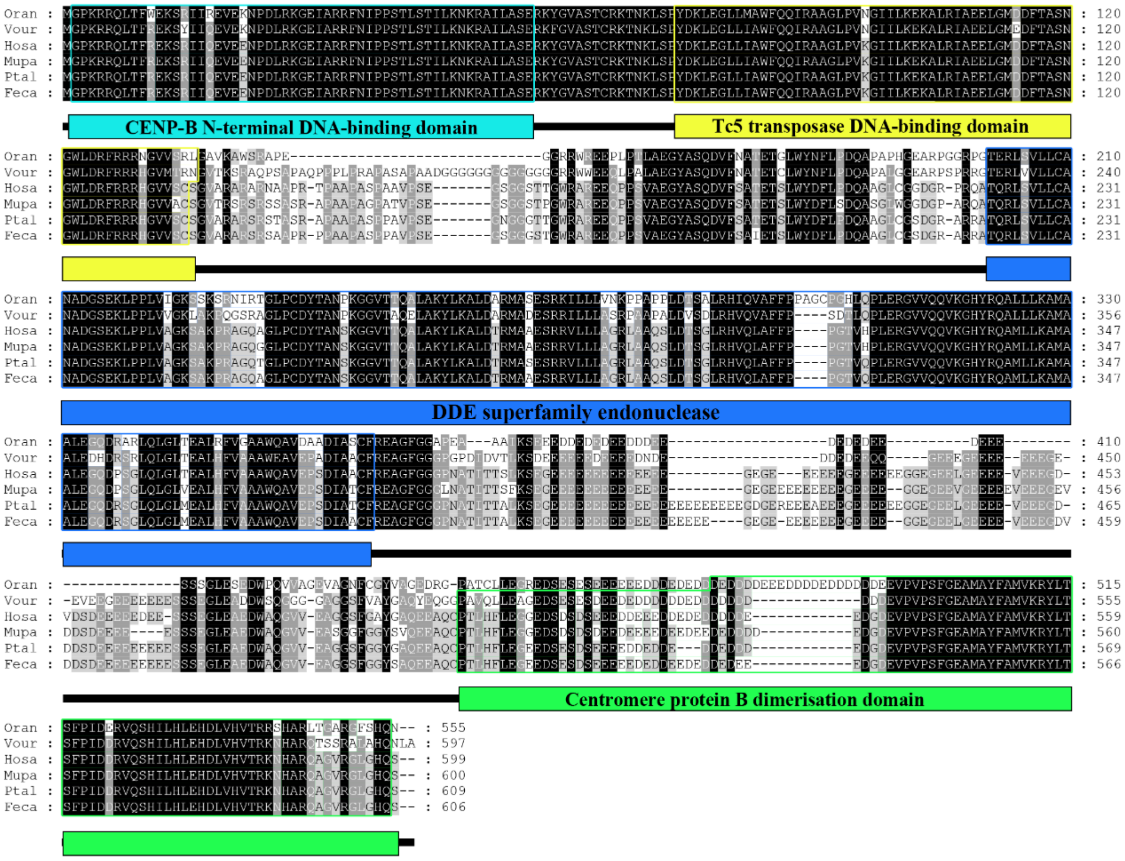

Figure S6A. Alignment of CENPB proteins. The Latin abbreviation of the species. Oran: Ornithorhynchus anatinus; Vour: Vombatus ursinus; Hosa: Homo sapiens; Mupa: Mus pahari; Ptal: Pteropus alecto; Feca: Felis catus.

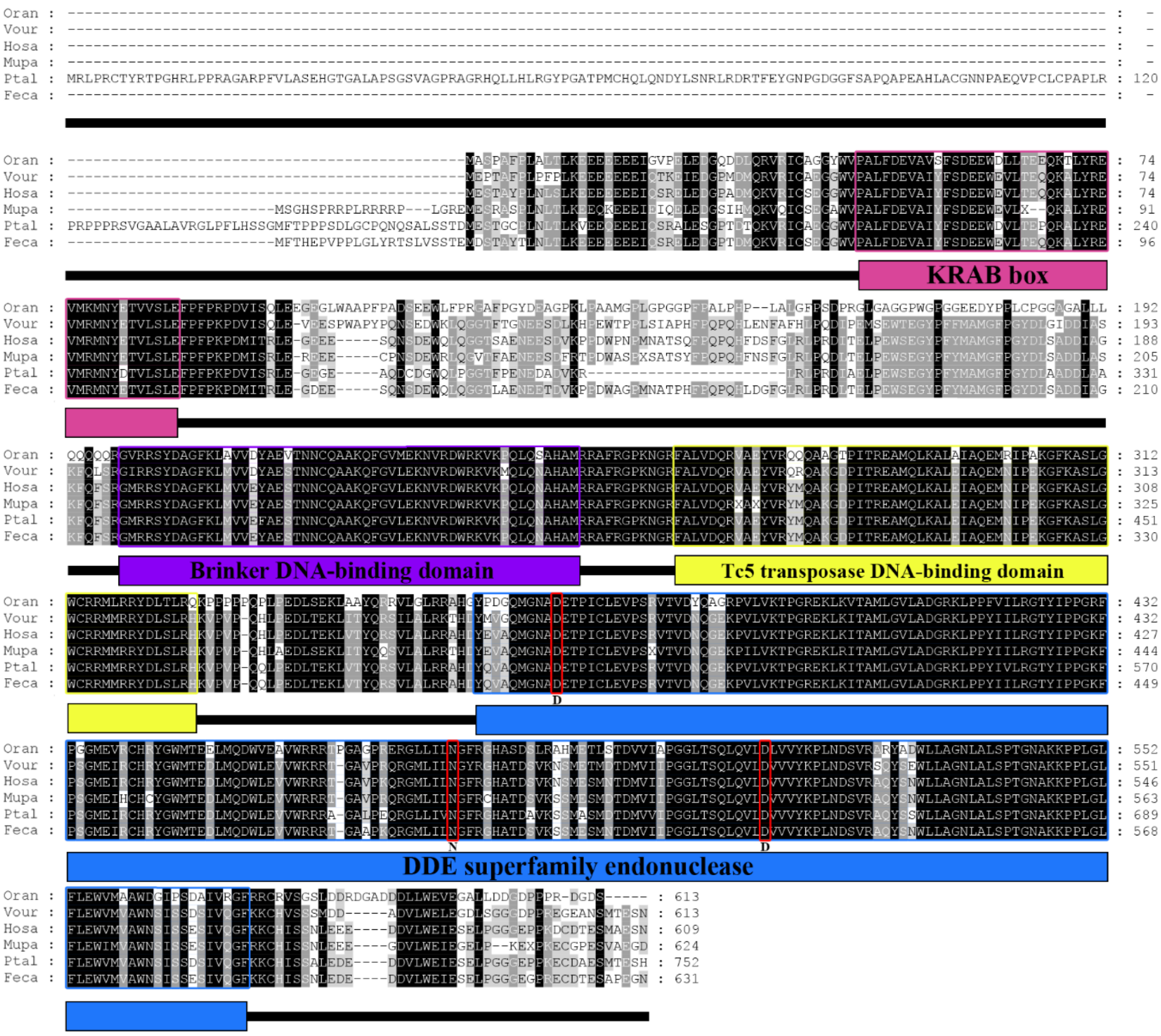

Figure S6B. Alignment of POGK proteins. The Latin abbreviation of the species. Oran: Ornithorhynchus anatinus; Vour: Vombatus ursinus; Hosa: Homo sapiens; Mupa: Mus pahari; Ptal: Pteropus alecto; Feca: Felis catus.

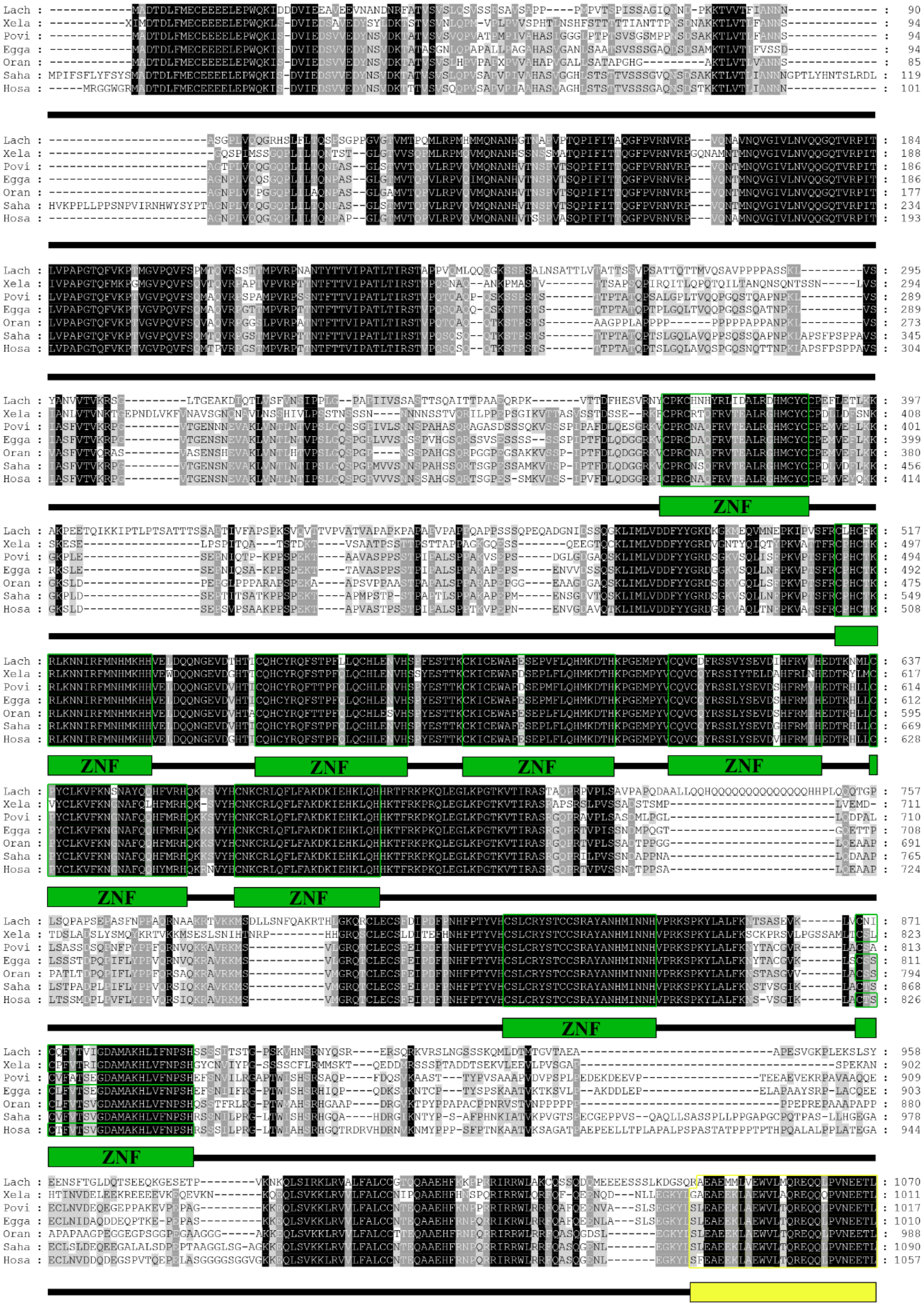
Lach
Xela
Povi
Egga
Oran
Saha
Hosa
ZNF

Figure continued

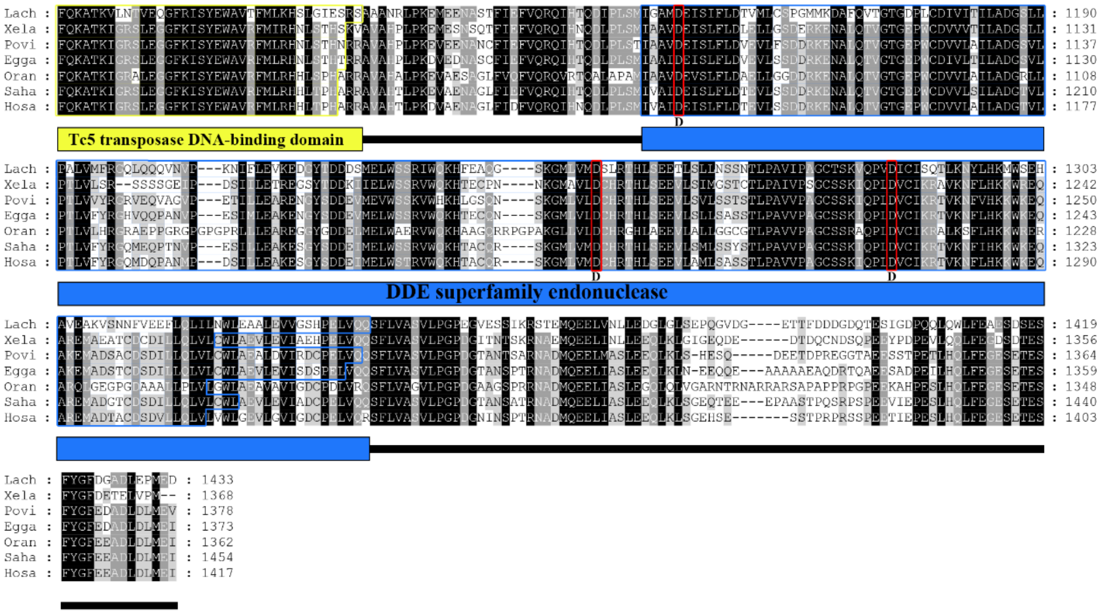

Figure S6C. Alignment of POGZ proteins. The Latin abbreviation of the species. Lach: Latimeria chalumnae; Xela: Xenopus laevis; Povi: Pogona vitticeps; Egga: Egretta garzetta; Oran: Ornithorhynchus anatinus; Saha: Sarcophilus harrisii; Hosa: Homo sapiens.

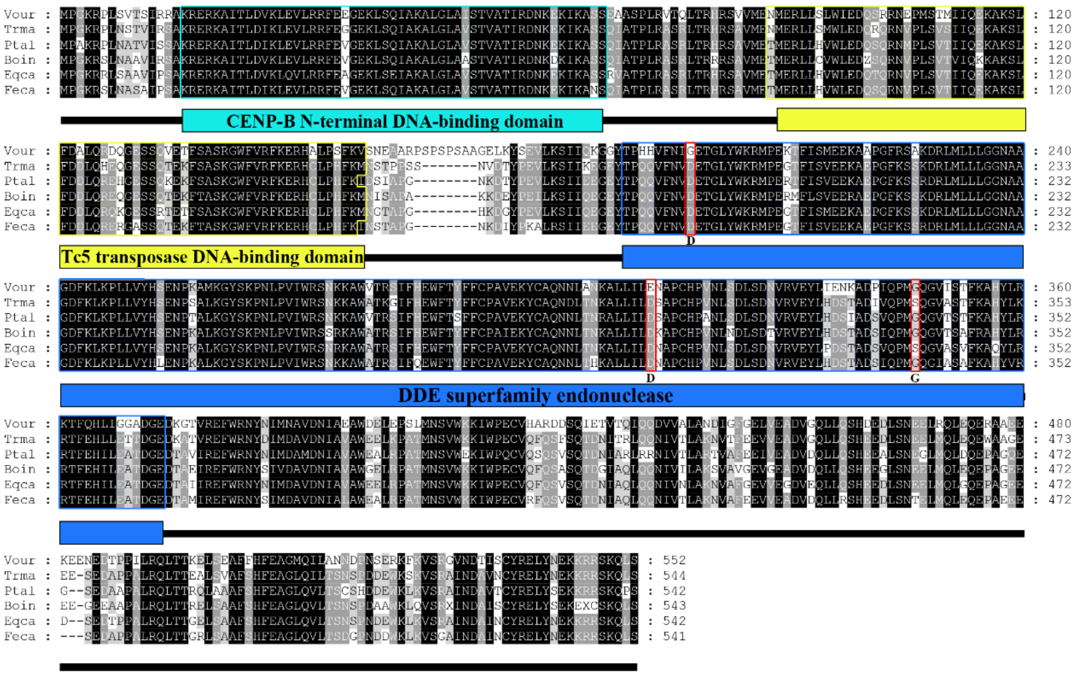

Figure S6D. Alignment of CENPBD1 proteins. The Latin abbreviation of the species. Vour: Vombatus ursinus; Trma: Trichechus manatus latirostris; Ptal: Pteropus alecto; Boin: Bos indicus; Eqca: Equus caballus; Feca: Felis catus.

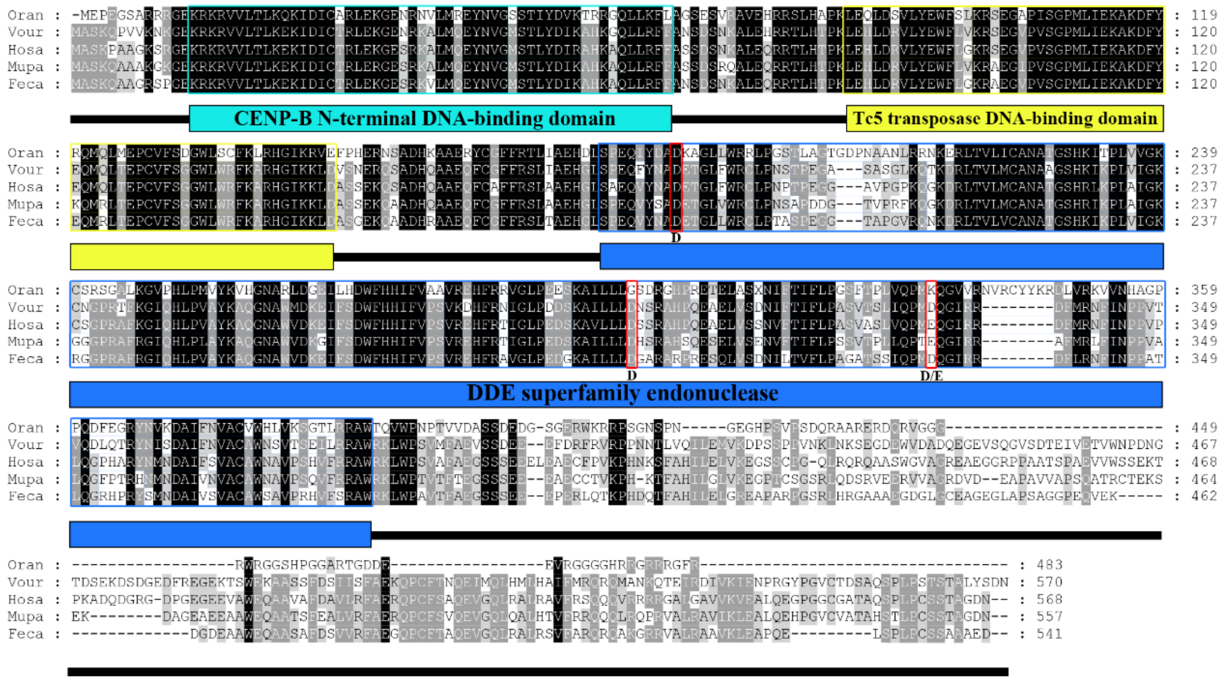

Figure S6E. Alignment of JRK proteins. The Latin abbreviation of the species. Oran: Ornithorhynchus anatinus; Vour: Vombatus ursinus; Hosa: Homo sapiens; Mupa: Mus pahari; Feca: Felis catus.

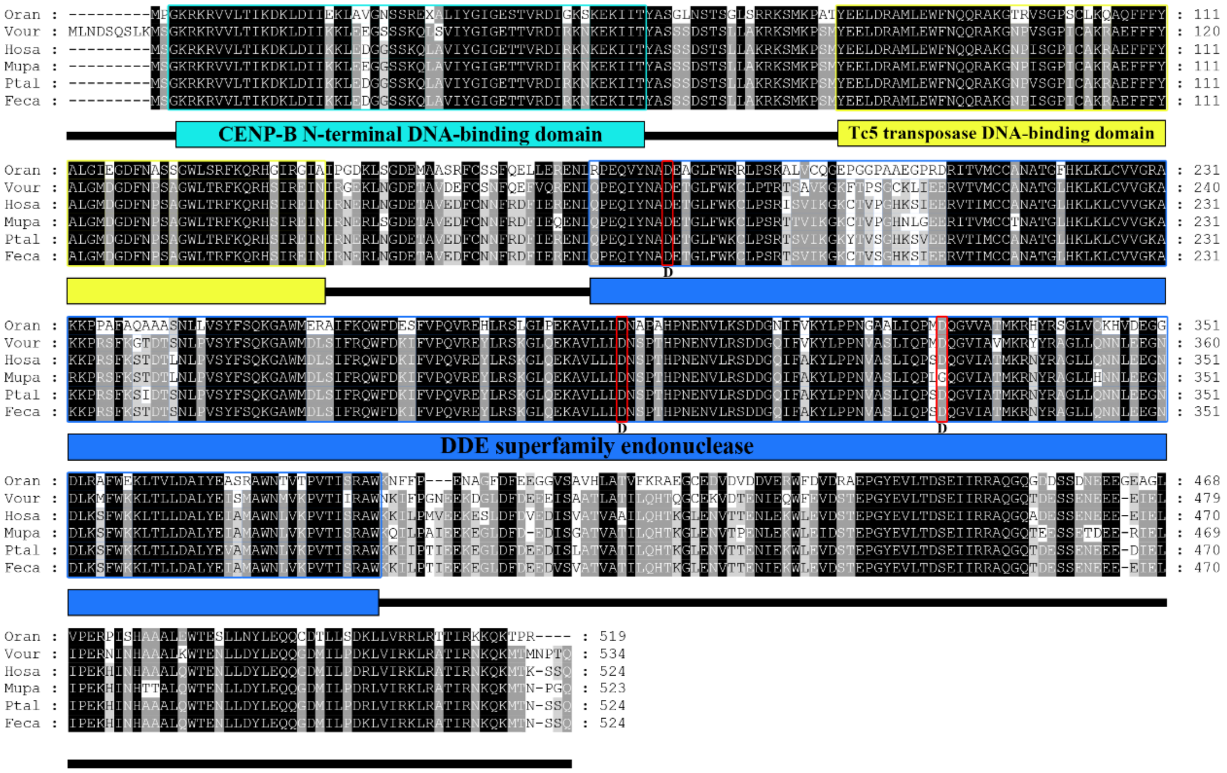

Figure S6F. Alignment of JRKL proteins. The Latin abbreviation of the species. Oran: Ornithorhynchus anatinus; Vour: Vombatus ursinus; Hosa: Homo sapiens; Mupa: Mus pahari; Ptal: Pteropus alecto; Feca: Felis catus.

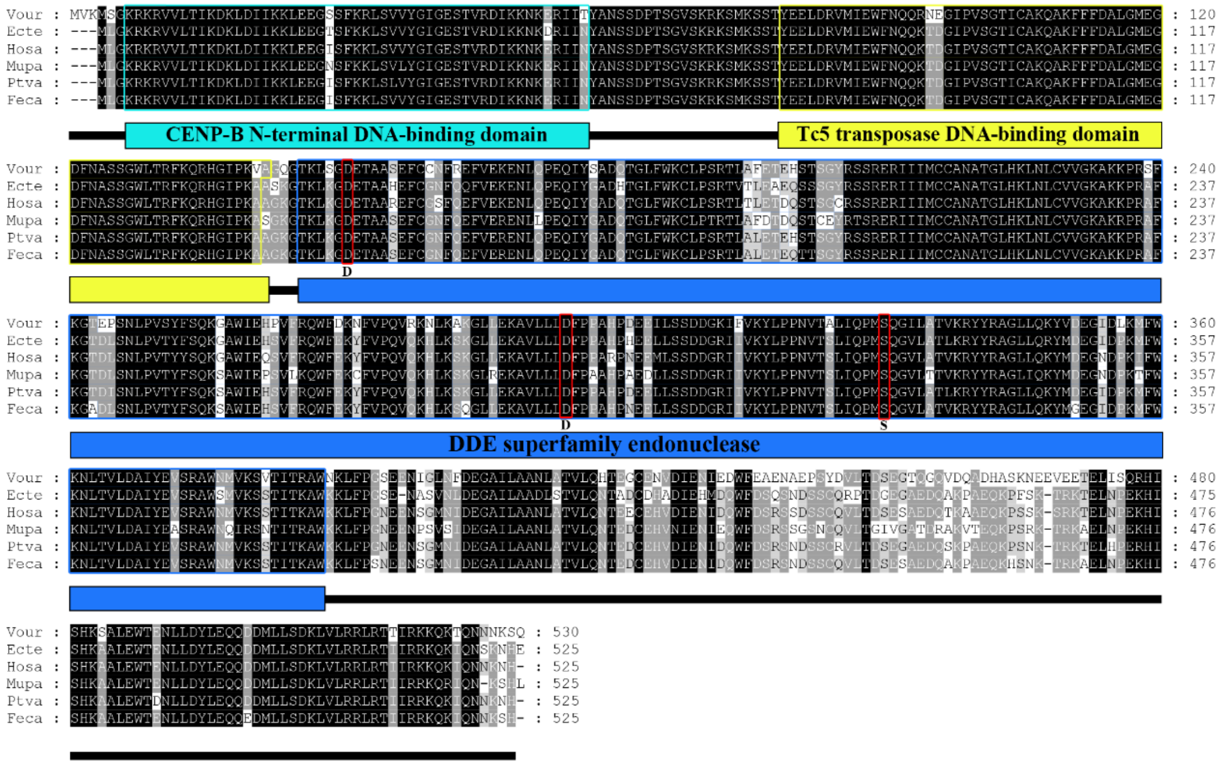

Figure S6G. Alignment of TIGD2 proteins. The Latin abbreviation of the species. Vour: Vombatus ursinus; Ecte: Echinops telfairi; Hosa: Homo sapiens; Mupa: Mus pahari; Ptva :Pteropus vampyrus; Feca: Felis catus.

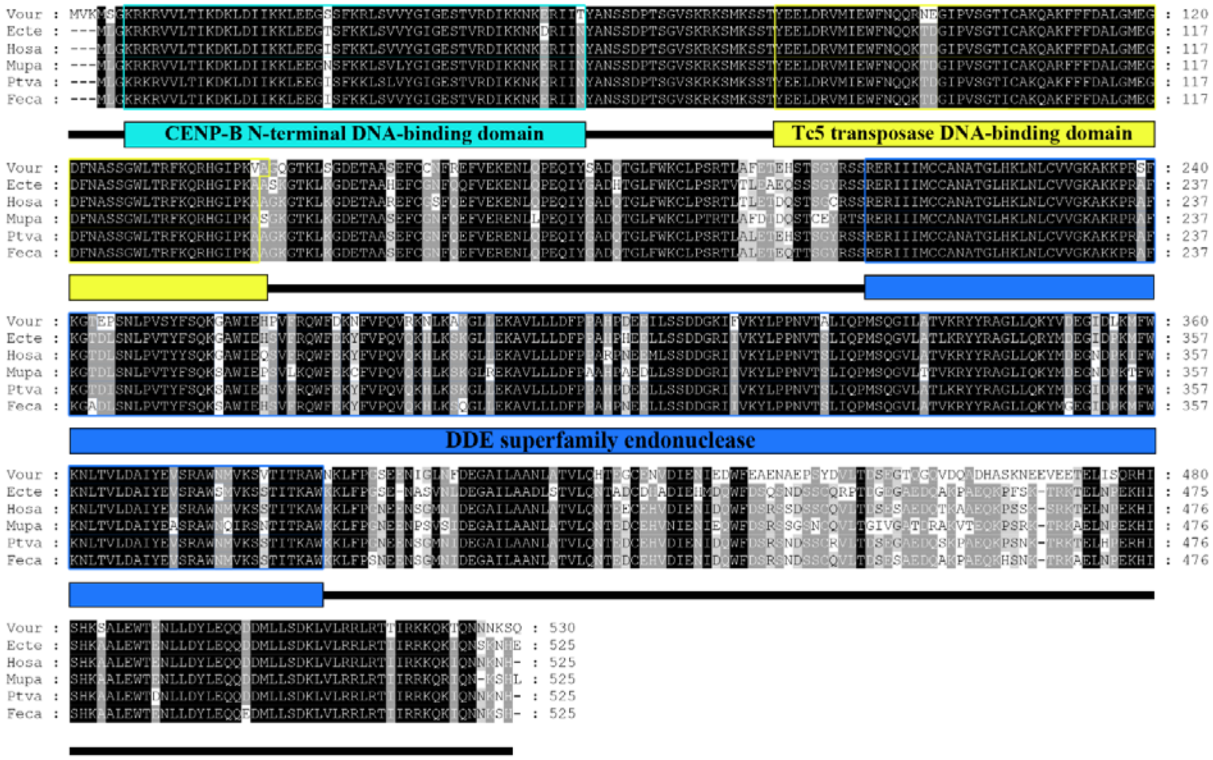

Figure S6H. Alignment of TIGD3 proteins. The Latin abbreviation of the species.Vour: Vombatus ursinus;Ecte: Echinops telfairi ; Hosa: Homo sapiens; Mupa: Mus pahari;Ptva Pteropus vampyrus; Feca: Felis catus.

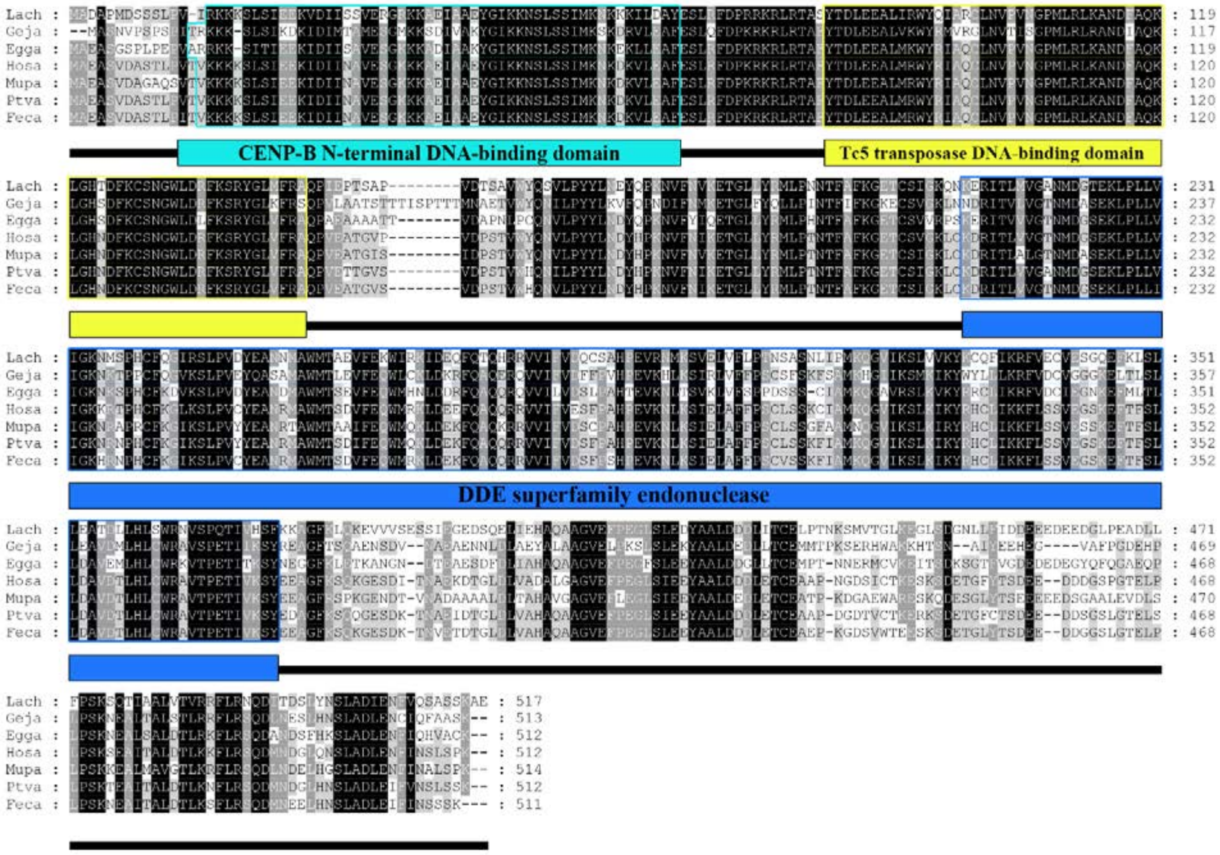

Figure S6I. Alignment of TIGD4 proteins. The Latin abbreviation of the species. Lach: Latimeria chalumnae; Geja: Gekko japonicus; Egga: Egretta garzetta ; Hosa: Homo sapiens; Mupa: Mus pahari; Ptva: Pteropus vampyrus; Feca: Felis catus.

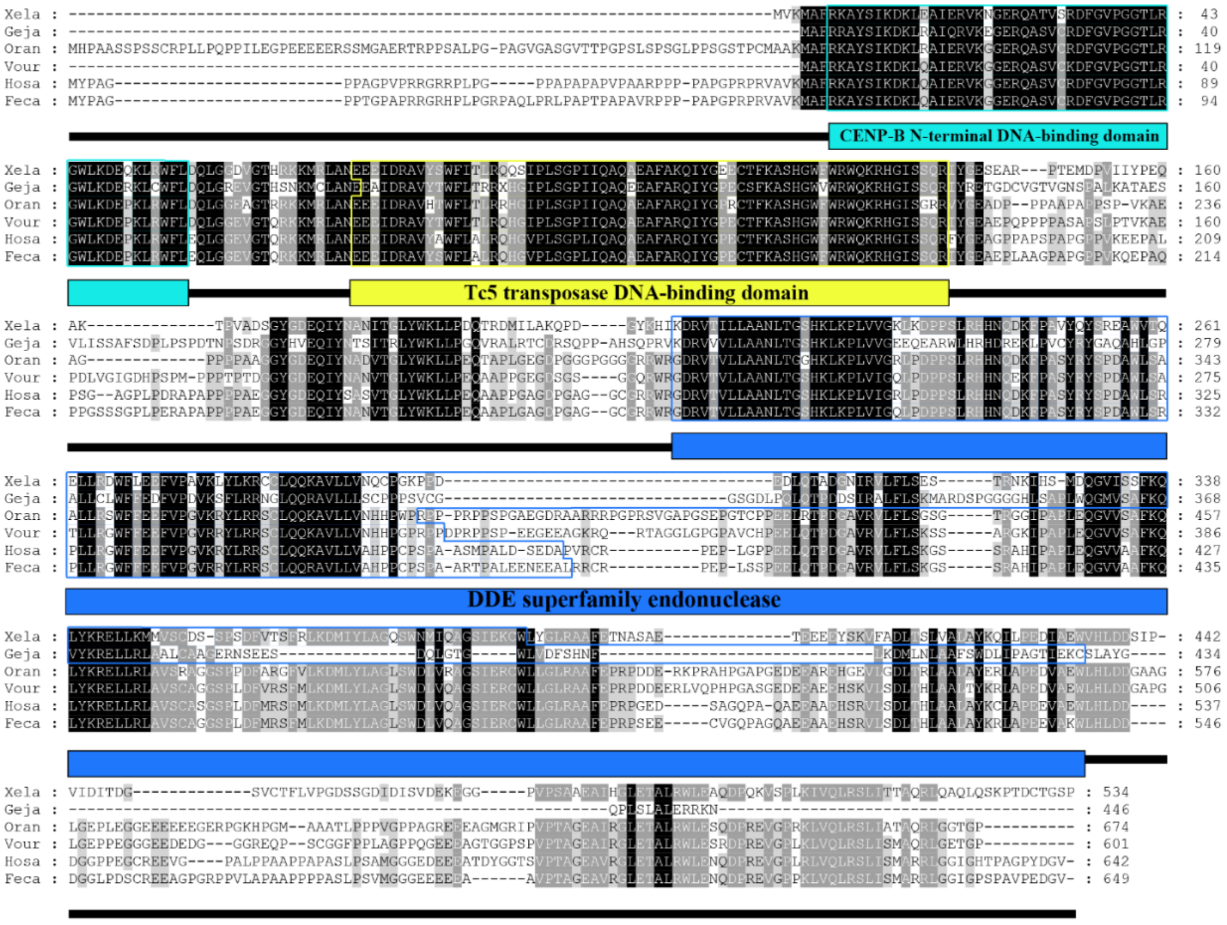

Figure S6J. Alignment of TIGD5 proteins. The Latin abbreviation of the species. Xela: Xenopus laevis; Geja: Gekko japonicus; Oran: Ornithorhynchus anatinus; Vour: Vombatus ursinus; Hosa: Homo sapiens; Feca: Felis catus.

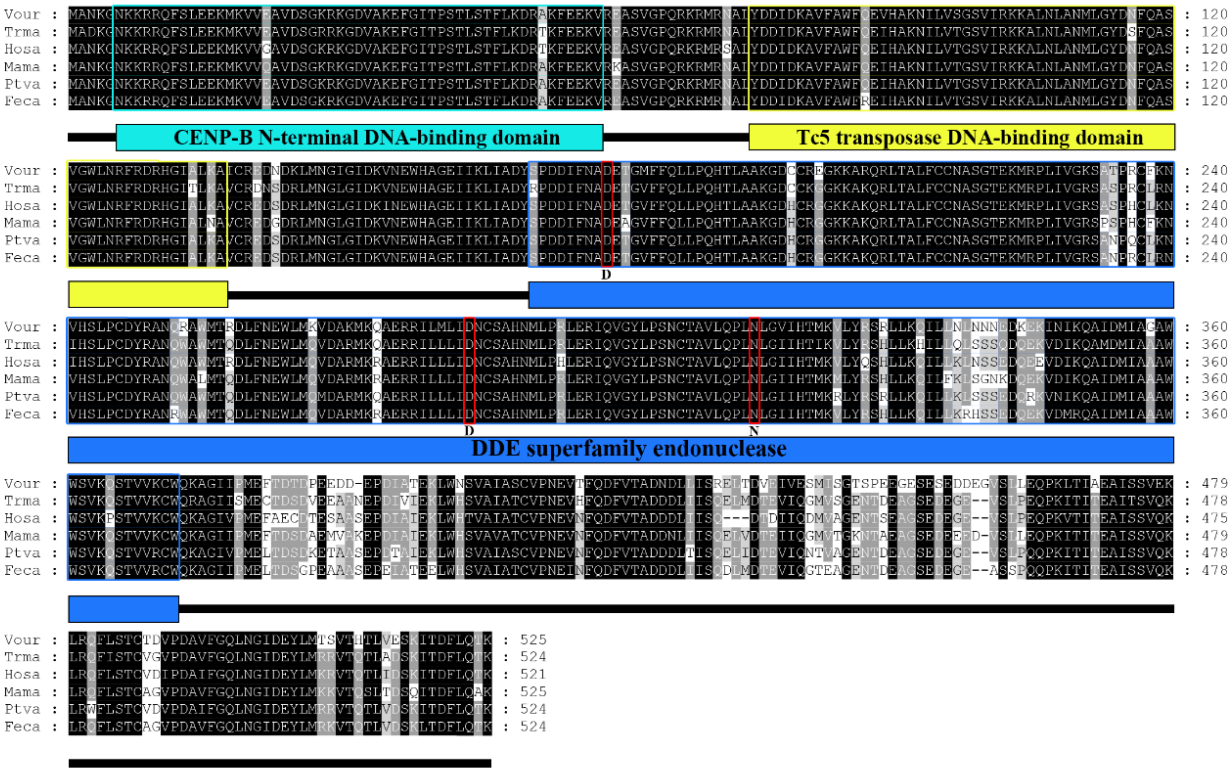

Figure S6K. Alignment of TIGD6 proteins. The Latin abbreviation of the species.Vour: Vombatus ursinus; Trma: Trichechus manatus latirostris; Hosa: Homo sapiens; Mama: Marmota marmota marmota; Ptva: Pteropus vampyrus; Feca: Felis catus.

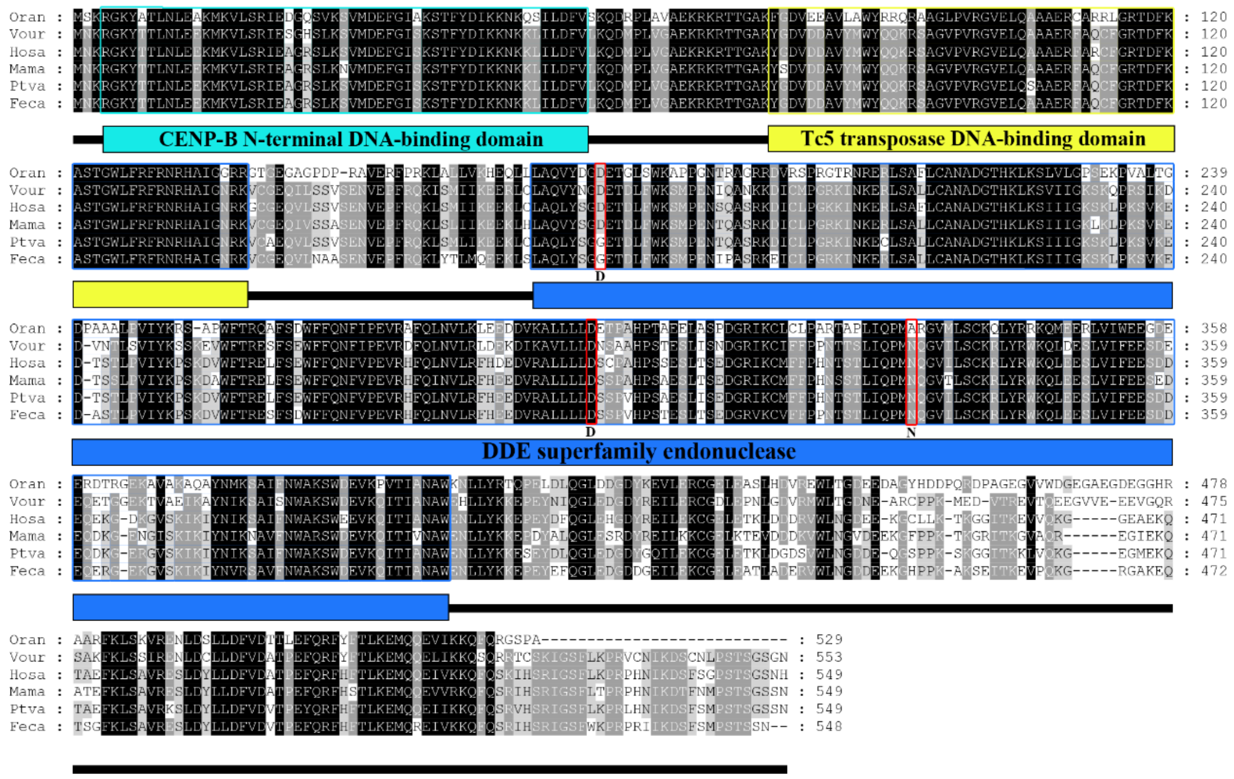

Figure S6L. Alignment of TIGD7 proteins. The Latin abbreviation of the species. Oran: Ornithorhynchus anatinus; Vour: Vombatus ursinus; Hosa: Homo sapiens; Marmota marmota marmota; Ptva: Pteropus vampyrus; Feca: Felis catus.
